# DNA damage-induced transcription stress triggers the genome-wide degradation of promoter-bound Pol II

**DOI:** 10.1038/s41467-022-31329-w

**Published:** 2022-06-24

**Authors:** Barbara Steurer, Roel C. Janssens, Marit E. Geijer, Fernando Aprile-Garcia, Bart Geverts, Arjan F. Theil, Barbara Hummel, Martin E. van Royen, Bastiaan Evers, René Bernards, Adriaan B. Houtsmuller, Ritwick Sawarkar, Jurgen Marteijn

**Affiliations:** 1 Department of Molecular Genetics, Oncode Institute, Erasmus MC Cancer Institute, Erasmus University Medical Center, Rotterdam, The Netherlands; 2 Max Planck Institute of Immunobiology and Epigenetics, Freiburg, Germany; 3 Department of Pathology, Optical Imaging Centre, Erasmus MC, Rotterdam, The Netherlands; 4 Oncode Institute, Division of Molecular Carcinogenesis, The Netherlands Cancer Institute, Amsterdam, The Netherlands; 5 MRC, University of Cambridge, Cambridge, UK

**Keywords:** Confocal microscopy, CRISPR-Cas9 genome editing, DNA damage response, Transcription

## Abstract

The precise regulation of RNA Polymerase II (Pol II) transcription after genotoxic stress is crucial for proper execution of the DNA damage-induced stress response. While stalling of Pol II on transcription-blocking lesions (TBLs) blocks transcript elongation and initiates DNA repair in cis, TBLs additionally elicit a response in trans that regulates transcription genome-wide. Here we uncover that, after an initial elongation block in cis, TBLs trigger the genome-wide VCP-mediated proteasomal degradation of promoter-bound, P-Ser5-modified Pol II in trans. This degradation is mechanistically distinct from processing of TBL-stalled Pol II, is signaled via GSK3, and contributes to the TBL-induced transcription block, even in transcription-coupled repair-deficient cells. Thus, our data reveal the targeted degradation of promoter-bound Pol II as a critical pathway that allows cells to cope with DNA damage-induced transcription stress and enables the genome-wide adaptation of transcription to genotoxic stress.

## Abstract (web summary)

DNA damage inhibits elongating RNA polymerase II, but also initiates genome-wide transcriptional responses. Here the authors reveal that particularly promoter-bound Pol II is degraded upon DNA damage in a GSK3 signaling-mediated response.

## Introduction

Transcription by RNA polymerase II (Pol II) is tightly regulated by a plethora of factors that control the binding of Pol II to promoters during initiation, and its subsequent release of the promoter proximal pause site into productive elongation^1,2^. Accurate transcription is constantly challenged by DNA damage that may cause transcription blocking lesions (TBLs), which hinder or fully block forward translocation of Pol II. Such Pol II obstructions by TBLs reduce or alter the pool of newly synthesized RNA molecules, causing severe cellular dysfunction^3–5^. If not repaired, TBLs may eventually lead to senescence or apoptosis and will ultimately contribute to DNA damage-induced aging^6^. To counteract these detrimental consequences of transcription stress, cells have evolved an intricate network of highly regulated processes to coordinate DNA repair, damage signaling and transcriptional output^4,7^. Central to this response is the dedicated repair pathway transcription-coupled nucleotide excision repair (TC-NER), which specifically removes TBLs from the transcribed strand of active genes. TC-NER is initiated when TBL-stalled Pol II is stably bound by Cockayne syndrome protein B (CSB)^8,9^, which leads to the successive recruitment of CSA^10^, and UV-stimulated scaffold protein A (UVSSA)^11^. This TC-NER complex is then recognized by the transcription factor IIH^12,13^, which unwinds the DNA and together with XPA verifies the TBL. Subsequently the endonucleases XPF/ERCC1 and XPG excise the damaged DNA, and the resulting single-strand gap is filled by DNA synthesis, and sealed by DNA ligases^14^. To allow access of this repair machinery to TBLs, it is assumed that lesion-stalled Pol II needs to be displaced. The currently most widely acknowledged model to achieve this involves backtracking of Pol II^5^. Alternatively, lesion-stalled Pol II may be dissociated during the repair reaction, or if TC-NER is limited, Pol II may be degraded to prevent the accumulation of persistently-stalled Pol II^15–17^.

The physical stalling of elongating Pol II on TBLs has long been acknowledged as the main cause for the overall reduced transcription rates upon UV^9,18–21^. However, accumulating evidence suggests that in addition to this stalling in cis, TBLs furthermore initiate a transcriptional response in trans, that regulates also non-stalled Pol II genome-wide^4,6^. This response in trans includes the repression of Pol II initiation^22–24^, the activation of Pol II pause release^25–27^, and the modulation of splicing^28–31^. Furthermore, the ubiquitylation of Pol II on lysine 1268 was reported to connect TBL-stalling to the regulation of transcription initiation by modulating Pol II stability^32^. These findings reveal that TBLs can have diverse effects on the regulation of Pol II transcription that are spatially separated from the TBL, but our understanding of such transcriptional regulation in trans and its contribution to the overall cellular response to DNA damage-induced transcription stress remains incomplete. Moreover, transcription responses in trans might have escaped their discovery because they are easily overshadowed by direct stalling of Pol II on TBLs (in cis), which is the prevailing response at high damage loads used in most experimental setups. To overcome this, we have recently developed a GFP-RPB1 knock in (KI) cell line, expressing endogenous levels of the largest subunit of Pol II tagged with GFP, as a sensitive tool to study Pol II transcription in living cells^33^. Here we applied this tool to discern the consequences of low dose UV irradiation on Pol II transcription. Our results uncovered that in addition to the UV-induced block of elongating Pol II in cis, TBLs also lead to the genome-wide proteasomal degradation of P-Ser5-modified promoter-bound Pol II in trans. This degradation, which is mediated by the ubiquitin-selective segregase VCP and signaled via GSK3, represents a DNA repair-independent mechanism to down-regulate Pol II transcription after UV, which helps cells to cope with the detrimental effects of TBLs.

## Results

### UV-induced DNA damage leads to loss of promoter-bound Pol II

**Fig. 1:**
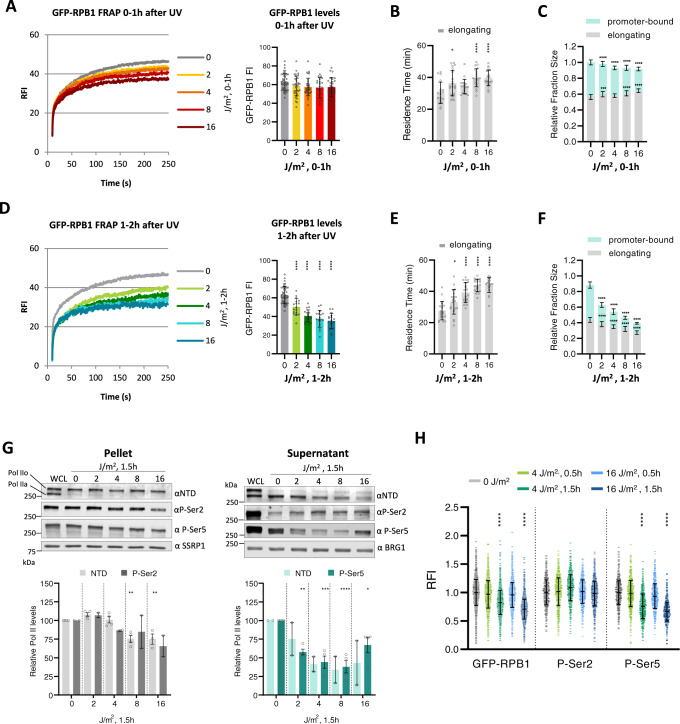
UV-induced DNA damage results in loss of promoter-bound Pol II. **a** Left panel: Fluorescence Recovery after photo-bleaching (FRAP) analysis of GFP-RPB1 in unperturbed conditions (0 J/m^2^), or within the first hour after irradiation with the indicated UV doses. GFP-RPB1 was bleached and fluorescence intensity was measured every 0.4 sec for 4 min, background-corrected and normalized to prebleach fluorescence intensity (FI), which was set to 100. RFI = relative FI. Right panel: Mean ± SD of pre-bleach GFP-RPB1 FI as a measure for Pol II protein levels in nuclei analyzed by FRAP. Cells from 4 independent experiments were analyzed. 0 J/m^2^ *n* =  *44*, 2 J/m^2^ *n* = 35 4 J/m^2^ *n* =  *33*, 8 J/m^2^ *n* = *26*, 16 J/m^2^ *n* = *27*. **b** Residence time of elongating Pol II obtained from Monte Carlo-based modeling of GFP-RPB1 FRAP data shown in A. Mean ±SD of the 20 best fitting simulations. **c** Relative size of the indicated Pol II fractions obtained from Monte Carlo-based modeling of GFP-RPB1 FRAP data shown in A. Mean ±SD of the 20 best fitting simulations. (**d**–**f**) Same as in A, B, and C, respectively, but between 1 and 2 h after UV irradiation. Cells from two independent experiments were analyzed. 0 J/m^2^ *n* =  44, 2 J/m^2^ *n* = 17, 4 J/m^2^ *n* =  17, 8 J/m^2^ *n* = 17, 16 J/m^2^ *n* = 17 cells for figure D, *n* = 20 simulations for figure E and F. **g** Top panel: Representative western blots after cellular fractionation of GFP-RPB1 KI cells 1 h after irradiation with the indicated UV doses. Cellular fractionation was optimized to separate hyper-phosphorylated, elongating Pol IIo in the pellet from hypo-phosphorylated, free and promoter-bound Pol IIa in the supernatant. SSRP1 and BRG1 were used as loading controls. WCL = whole cell lysate, NTD = RPB1 N-terminal domain, P-Ser= phospho-serine. Bottom panel: Quantification of the indicated western blot signals, *n* = 2 for P-Ser2 and NTD of supernatant fraction, *n* = 4 for P-Ser5 and NTD of pellet fraction, mean ± SE. **h** Total Pol II levels determined by GFP-RPB1 fluorescent signal, and P-Ser2 and P-Ser5 levels determined by immunofluorescence in GFP-RPB1 KI cells by high-content microscopy. Relative fluorescence intensities (RFI) were normalized to 0 J/m^2^ levels which was set to 1. *n* = 4531, 3182, 3350, 3365, 4042, 1462, 1268, 1261, 1117, 1400, 1514, 1212, 1314,1133, 1408 cells (left to right), error bars represent SD. *P ≤  0.05, **P ≤  0.01, ***P ≤  0.001, ****P ≤  0.0001; analyzed by ordinary one-way ANOVA using Dunnett’s multiple comparisons test versus the 0 J condition (**a**–**h**).

To study the effects of UV-induced DNA damage on Pol II transcription, we used fluorescence recovery after photo-bleaching (FRAP)^34,35^ of endogenously-expressed GFP-RPB1, a sensitive tool to identify perturbations of transcription at the different stages of the transcription cycle^33^. Promoter-bound Pol II are chromatin-bound for less than a minute during initiation or promoter-proximal pausing, and therefore mostly contribute to the fast, initial recovery of fluorescence during FRAP (<50 sec) (Fig. 1a). In contrast, elongating Pol II are chromatin-bound for >20 min on average, and mainly represent the later part of the GFP-RPB1 FRAP curve (>100 sec)^33^. Of note, GFP-RPB1 knock-in did not affect transcription^33^ or the DNA damage-induced transcription response, as the recovery of RNA synthesis (Supplementary Fig. 1a) and clonogenic survival (Supplementary Fig. 1b) after UV-damage was similar to wild-type cells.

To study the consequences of TBLs on Pol II transcription we irradiated GFP-RPB1 KI cells with increasing doses of UV and analyzed Pol II protein levels and chromatin binding kinetics in real time (Fig. 1a). UV doses were chosen so that at the highest dose (16 J/m^2^) all transcribed gene strands are expected to contain at least one TBL^36^, while at the lowest dose (2 J/m^2^) most genes remain undamaged, allowing observation of DNA damage-induced effects on transcription in trans. Of note, cells showed full recovery of transcription up to a UV dose of 8 J/m^2^ (Supplementary Fig. 1c).

Within the first hour after UV irradiation, we observed a dose-dependent immobilization of Pol II. This immobilization was particularly evident from the reduced slope of the FRAP curve at time points >100 sec (Fig. 1a, left panel), which mainly represents elongating Pol II. Interestingly, GFP-RPB1 fluorescence, a direct measure for Pol II protein levels, remained unchanged within the first hour after UV irradiation (Fig. 1a, right panel), indicating that under these conditions lesion-stalled Pol II was not degraded. While inhibition of transcription initiation (THZ1) or pause release (Flavopiridol) resulted in mobilization of Pol II, treatment with Pol II elongation inhibitors α-Amanitin and Cordycepin induced a similar Pol II immobilization as observed upon exposure to UV (Supplementary Fig. 1d)^33,37,38^, strongly indicating that the DNA damage-induced Pol II immobilization is explained by reduced Pol II elongation rates. To quantitatively interpret Pol II kinetics after UV damage, we computationally simulated GFP-RPB1 FRAP curves by Monte Carlo (MC)-based modeling, and fitted the simulated curves to the Pol II Strip-FRAP curves. This approach allows to extract the average fraction sizes and chromatin binding times of the kinetically distinct promoter-bound and elongating Pol II populations^33,39^. We found that within the first hour after UV irradiation the fraction of elongating Pol II slightly increased (Fig. 1c), and that the average chromatin binding of elongating Pol II was prolonged by up to 25% (Fig. 1b), indicating that there is more elongating Pol II with a slower elongation rate. This dose-dependent reduction in elongation speed can most likely be explained by the direct, physical stalling of a subset of elongating Pol II on TBLs, which increases at higher UV doses^9,19,20,31^. This UV dose-dependent increase in residence time of elongating Pol II without degradation indicates that most Pol II is not released from chromatin and replaced by other Pol II complexes, but remains prolonged chromatin-bound after UV-induced DNA damage.

In addition to the direct effect of UV on Pol II elongation (Figs. 1a), 1 hour after UV exposure FRAP experiments showed that Pol II was significantly further immobilized (Fig. 1d). Interestingly, this additional immobilization mainly affected the initial part of the FRAP curve (<50 sec), representing mostly promoter-bound Pol II^33^, while the slope of the later part of the FRAP curve (>100 sec) did not change between the first and the second hour after UV irradiation (Fig. 1d, left panel). These data indicate that 1 h after UV the Pol II elongation rate is not further reduced, but that specifically the fraction size of promoter-bound Pol II is strongly reduced. As total Pol II protein levels determined by GFP-RPB1 fluorescence were also reduced 1 h after UV irradiation (Fig. 1d, right panel), this suggests that the Pol II immobilization was caused by the degradation of promoter-bound Pol II. Indeed, MC modeling of Pol II kinetics^33^ 1 h after UV damage revealed a ~75% reduced fraction size of promoter-bound Pol II, while the fraction size of elongating Pol II was only marginally decreased (Fig. 1f). Furthermore, MC-modeling indicated that the residence time of elongating Pol II did not increase further compared to time-points directly after irradiation (Fig. 1b, e). Strikingly, and in contrast to the dose-dependent elongation block (Fig. 1b, e), the UV-induced loss of promoter-bound Pol II 1 h after irradiation was rather similar at UV doses ranging from 2 to 16 J/m^2^ (Fig. 1f), and thus did not correlate directly with the damage load. This suggests that the additional loss of mobile Pol II is most likely not caused by the direct stalling of Pol II on TBLs.

As the mobile fraction of Pol II determined by FRAP represents freely diffusing, as well as initiating and promoter-paused Pol II^33^, we next determined which of these Pol II fractions was lost 1 h after low dose UV irradiation. Therefore, we separated stably chromatin-bound, elongating Pol II from transiently promoter-bound^33,40–42^, initiating and promoter-paused Pol II using a previously described cell fractionation procedure^33^. Successful fractionation is illustrated by the clear separation of P-Ser2-modified Pol IIO (hyper-phosphorylated) in the pellet fraction, and Pol IIA (hypo-phosphorylated) in the supernatant fraction (Fig. 1g). P-Ser5-modified Pol II is specifically recognized by the phospho-specific antibody as this fraction is lost after CDK7 inhibition by THZ1^43^ (Supplementary Fig. 1e). In contrast, CDK9 inhibition by Flavopiridol, which inhibits Pol II release from the promoter-proximal pause site thereby increasing the fraction of promoter-paused Pol II^44,45^, enlarged the P-Ser5-modified Pol II fraction in the supernatant (Supplementary Fig. 1e). Therefore, the P-Ser5-modified Pol II in the supernatant fraction represents promoter-paused Pol II that is most likely released from the pellet fraction during the fractionation procedure^33^ due to its highly mobile nature^33,41,42^. Indeed, PFA-mediated crosslinking before fractionation retained P-Ser5-modified Pol II in the pellet fraction upon Flavopiridol treatment (Supplementary Fig. 1f).

While no obvious effects could be observed 15 min after UV, these fractionation experiments showed a clear reduction of P-Ser5-modified Pol II levels in the supernatant 90 min after UV irradiation (Fig. 1g and Supplementary Fig. 1g), which is in line with the dose-independent loss of mobile Pol II observed by FRAP analysis 1 h after UV (Fig. 1d, left panel). This corroborates that the reduced fluorescence recovery of Pol II 1 h after UV (Fig. 1d, right panel) is caused by a loss of promoter-bound Pol II. Importantly, the levels of elongating Pol II (P-Ser2 in the pellet) remained largely unaffected 1 h after low dose UV. Only after irradiation with a higher UV dose (16 J/m^2^) the levels of elongating Pol II were slightly reduced, which may be explained by a chromatin release of elongating Pol II, which is in line with reduced decrease of P-Ser5 levels in the supernatant at 16 J/m^2^ compared to UV doses of 2–8 J/m^2^ (Fig. 1g).

The UV-induced loss of Pol II 1 h after 4 and 16 J/m^2^ of UV was also confirmed by high content immuno-fluorescence quantification (Fig. 1h). While the levels of total Pol II (GFP) and P-Ser5-modified Pol II were reduced to a highly similar extent after 4 J/m^2^ of UV, elongating Pol II (P-ser2) levels remained largely unaffected. Together these results show that the dose-independent loss of mobile Pol II observed by FRAP is caused by a specific loss of P-Ser5-modified Pol II after the initial elongation block.

### Promoter-bound Pol II is degraded after UV-induced DNA damage

**Fig. 2:**
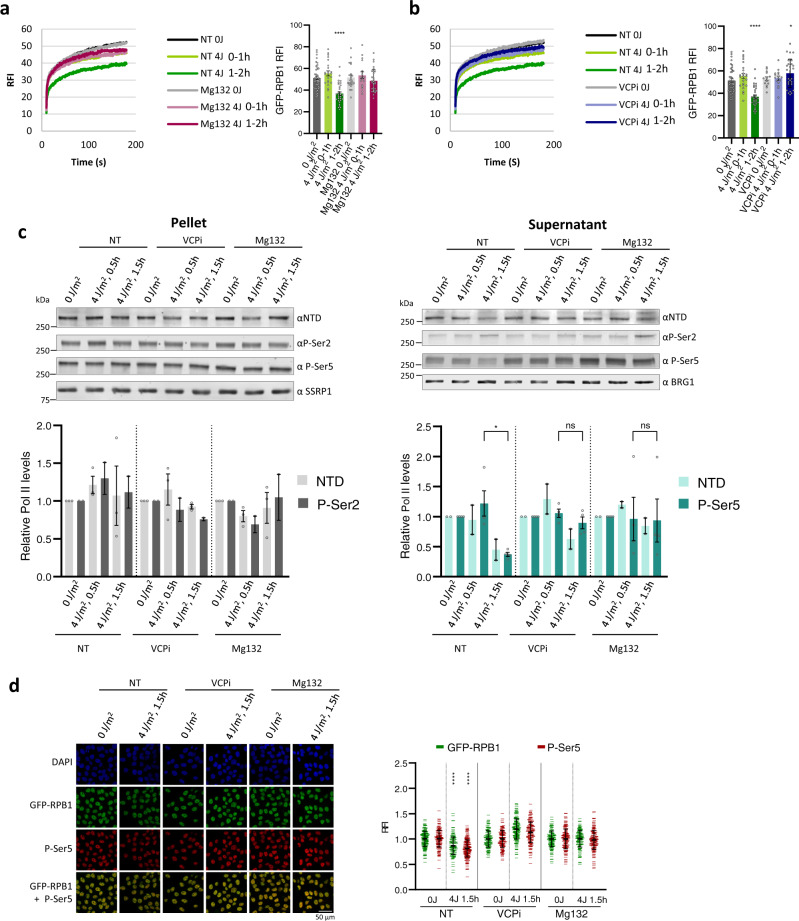
UV-induced proteasomal degradation of promoter-bound Pol II. **a**, **b** Left panel: Fluorescence Recovery after photo-bleaching (FRAP) of GFP-RPB1 after UV irradiation with or without pre-treatment for 30 min with (**a**) proteasome inhibitor (Mg132, 10 µM) or (**b**) VCP inhibitor (VCPi) (NMS-873, 5 µM). GFP-RPB1 was bleached and fluorescence intensity was measured every 0.4 sec for 3 min, background-corrected and normalized to prebleach fluorescence intensity (FI), which was set to 100. RFI = relative FI. Right panel: Mean ±SD of pre-bleach GFP-RPB1 FI as a measure for Pol II protein levels in nuclei analysed by FRAP. Cells from two independent experiments were analyzed. 0 J/m^2^ *n* =  42, 4 J/m^2^ 0–1 h *n* = 24, 4 J/m^2^ 1–2 h *n* =  30, MG132 0 J/m^2^ *n* = 22, MG132 4 J/m^2^ 0–1 h *n* = 16, MG132 4 J/m^2^ 1–2 h *n* = 29, VCPi 0 J/m^2^ *n* = 14, VCPi 4 J/m^2^ 0–1 h *n* = 16, VCPi 4 J/m^2^ 1–2 h *n* = *28*. (**c**) Top panel: Representative western blots after cellular fractionation of GFP-RPB1 knock in (KI) cells after the indicated treatments. SSRP1 and BRG1 were used as loading controls. WCL = whole cell lysate, NTD = RPB1 N-terminal domain, P-Ser= phospho-serine. Bottom panel: Quantification of the indicated western blot signals, NTD pellet fraction *n* = 3, Ser2 pellet fraction and NTD supernatant *n* = 2, P-Ser5 *n* = 4, mean ± SE. **d** Left panel: Representative images of GFP-RPB1 KI cells that were fixed and stained for P-Ser5 after UV irradiation without or with pre-treatment for 30 min with proteasome inhibitor (Mg132, 10 µM) or VCP inhibitor (VCPi) (NMS-873, 5 µM). Right panel: GFP-RPB1 and P-Ser5 fluorescence intensities (FI) were normalized were normalized to 0 J/m^2^ levels, which were set to 1. RFI Relative FI. *n* > 200 cells, error bars represent SD. *P ≤  0.05, ****P ≤  0.0001, ns nonsignificant; analyzed by ordinary one-way ANOVA using Dunnett’s multiple comparisons test versus the respective 0 J condition (**a**–**d**).

Elongating Pol II has been shown to be targeted for degradation by the 26 S proteasome after DNA damage^46–53^. To investigate whether also the UV-induced loss of promoter-bound Pol II is caused by proteasomal degradation, we determined Pol II protein levels and kinetics in the presence of the proteasome inhibitor Mg132 (Fig. 2a). Proteasome inhibition fully rescued the UV-induced Pol II loss quantified by GFP-RPB1 live-cell fluorescence (Fig. 2a and Supplementary Fig. 2a, right panels), and fully rescued the additional immobilization of Pol II 1 h after UV (Fig. 2a and Supplementary Fig. 2a, left panels). Together, this suggests that promoter-bound Pol II is degraded by the 26 S proteasome and that this degradation causes the Pol II immobilization 1 h after the initial elongation block. Proteasomal degradation of elongating Pol II has been shown to be dependent on the ubiquitin-specific segregase VCP/p97^54–57^. To test whether also P-Ser5-modified Pol II is degraded in a VCP-dependent manner, we studied the effects of VCP inhibition (VCPi) on UV-induced Pol II degradation. Similar to proteasome inhibition, VCP inhibition prior to UV irradiation also rescued Pol II protein levels and mobility (Fig. 2b and Supplementary Fig. 2b). Interestingly, proteasome or VCP inhibition did not affect the immobilization of elongating Pol II, i.e. the FRAP measurement at time points >100 s, suggesting that under these conditions promoter-bound Pol II is the primary VCP substrate, and not lesion-stalled Pol II. Cellular fractionation experiments (Fig. 2c) confirmed that rescue of Pol II protein levels after VCP or proteasome inhibition was mainly due to the rescue of P-Ser5 Pol II levels in the supernatant. The rescue of total and P-Ser5-modified Pol II by the proteasome or VCP inhibition was confirmed by quantifying the GFP-RPP1 fluorescence and P-Ser5-modified Pol II signal by immunofluorescence (Fig. 2d). As VCP and proteasome inhibition will rescue specifically the Pol II fraction that has been targeted for degradation, and elongating Pol II was not affected by inhibiting VCP, these results corroborate that UV irradiation predominantly leads to the degradation of a mobile, P-Ser5-modified promoter-bound Pol II fraction.

### P-Ser5-modified, promoter-bound Pol II is ubiquitylated and extracted by VCP for proteasomal degradation upon DNA damage

**Fig. 3:**
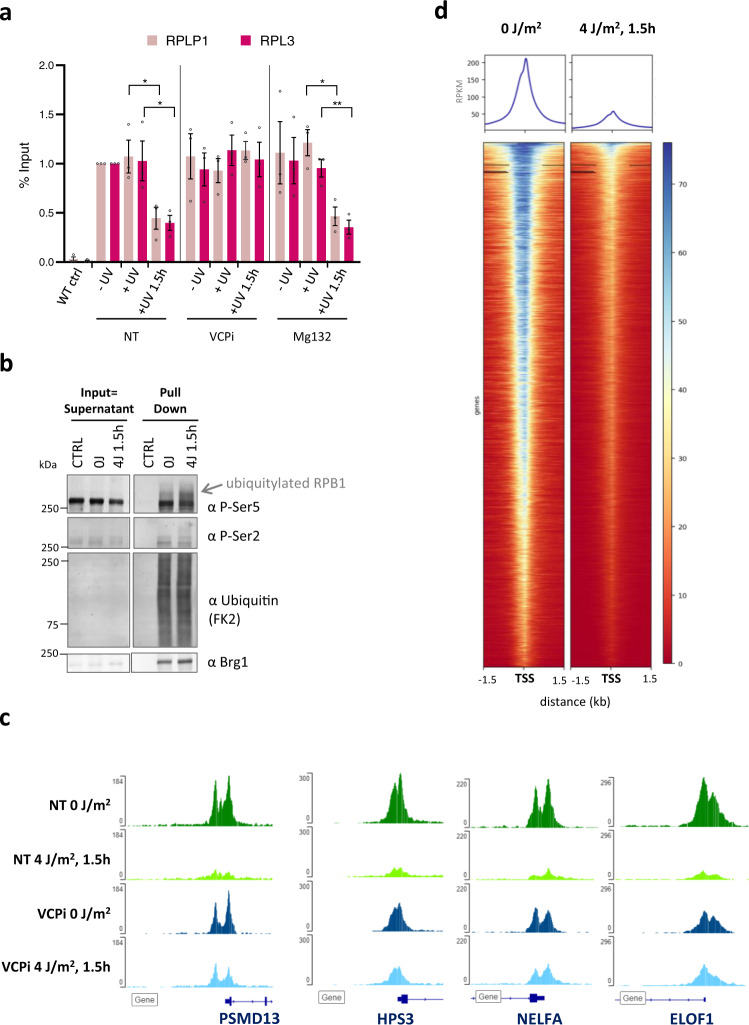
P-Ser5-modified, promoter-bound Pol II is ubiquitylated, extracted by VCP and subsequently degraded. **a** Pol II promoter occupancy on the RPLP1 and RPL3 genes in GFP-RPB1 KI cells after the indicated treatments determined by GFP ChIP followed by q-PCR. Wild-type cells without GFP-RPB1 were analysed as negative control (WT Ctrl). VCPi= VCP inhibitor (NMS-873, 5 µM 30 min before UV irradiation), Mg132=proteasome inhibitor (10 µM, 30 min before UV irradiation). Average Pol II occupancy around the transcription start site of *n* = 3 independent experiments is shown, error bars indicate SE. All conditions were normalized to promoter occupancy in nonperturbed conditions (NT -UV), which was set to 1. *P ≤  0.05, **P ≤  0.01, ns nonsignificant; analyzed by ordinary one-way ANOVA using Dunnett’s multiple comparisons test. **b** Isolation of ubiquitylated proteins using tandem ubiquitin-binding entities (TUBEs) from the supernatant of GFP-RPB1 KI cells after cellular fractionation. Cellular fractionation was optimized to separate hyper-phosphorylated, elongating Pol IIo in the pellet from hypo-phosphorylated, free and promoter-bound Pol IIa in the supernatant. Isolated ubiquitylated proteins were stained for promoter-paused Pol II (P-Ser5) and ubiquitin (FK2). FK2 staining confirms equal purification of ubiquitylated proteins. Brg1 staining was used as loading control. Note that ubiquitylated, P-Ser5 Pol II (pull-down) migrates higher compared to P-Ser5 Pol II in the input due to increased molecular weight. P-Ser = phospho-serine. The experiment has been performed two times with similar results. **c**, **d** Pol II occupancy around the transcription start site was analysed by Pol II ChIP-seq before and 1 h after 4 J/m^2^ UV, and with or without VCP inhibition before UV irradiation. Pol II signal at representative individual genes (**c**), and heatmaps of Pol II on all refseq genes before and 1 h after 4 J/m^2^ UV (**d**). Values were normalized to spike in controls. Heat maps are colour-scaled according to read densities form low (red) to high (blue) in units of RPM.

To investigate whether the VCP-mediated proteasomal degradation of P-Ser5-modified Pol II targets promoter-bound Pol II, we performed Pol II chromatin immunoprecipitation (ChIP) experiments and analyzed Pol II promoter occupancy on two individual genes by qPCR (Fig. 3a). In line with FRAP and fractionation results (Figs. 1–2**)**, ChIP-qPCR showed that in non-treated cells (NT) Pol II chromatin binding ~100 bp downstream of the transcription start site (TSS) of RPLP1 and RPL3 was not affected directly after UV, but was strongly reduced 1 h after UV. VCP inhibition fully rescued the UV-induced loss of Pol II promoter occupancy (Fig. 3a), in line with the rescue of promoter-bound Pol II protein levels (Fig. 2b, c). In contrast to VCP inhibition, proteasome inhibition did not rescue UV-induced loss of promoter occupancy, while it fully rescued the protein levels of promoter-bound Pol II as assessed in the supernatant fraction after fractionation (Fig. 2a, c). These results indicate that promoter-bound Pol II is extracted from chromatin by VCP prior to proteasomal degradation. As VCP is a ubiquitin-specific segregase, this implies that promoter-bound Pol II is ubiquitylated after low-dose UV irradiation. To test this, we purified endogenously ubiquitylated proteins using TUBEs^58^ after irradiation with 4 J/m^2^ (Fig. 3b). As also TBL-stalled elongating Pol II can be ubiquitylated^16^, we isolated ubiquitylated proteins specifically from the supernatant fraction, which contains negligible amounts of elongating Pol II, as shown by the absence of P-Ser2 signal in the input compared to the pellet fraction (Supplementary Fig. 3a). Ubiquitin staining showed successful and equal isolation of ubiquitylated proteins in non-treated (NT) and UV-irradiated (4 J/m^2^) samples. Importantly, P-Ser5 staining in the pull-down fraction showed increased levels of ubiquitylated, higher migrating Pol II after UV, demonstrating that P-Ser5-modified, promoter-bound Pol II is indeed ubiquitylated after UV irradiation.

To test the extent to which VCP mediates the degradation of promoter-bound Pol II we obtained genome-wide profiles of Pol II transcription start site (TSS) occupancy by Pol II ChIP-Seq after irradiation with UV. This revealed that 1.5 h after UV irradiation Pol II promoter-binding at the TSS is severely reduced, as seen on representative individual genes (Fig. 3c), and genome-wide by metagene analysis (Fig. 3d). In line with the qPCR results (Fig. 3a), this UV-induced reduction of Pol II promoter-binding was rescued by VCP inhibition (Supplementary Fig. 3b). Under the used conditions, VCP inhibition did not affect transcription rates in undamaged cells (Fig. 2b and Supplementary Fig. 3c). Interestingly, irradiation with 4 J/m^2^ generates a lesion density that theoretically leaves the majority of genes undamaged^36^. As promoter regions are relatively small compared to gene bodies, it is highly unlikely that all promoters contain a TBL. This indicates that the genome-wide VCP-dependent loss of Pol II promoter binding is not directly caused by TBLs in cis, but argues for a UV-induced regulation of promoter-bound Pol II in trans.

### The UV-induced degradation of promoter-bound Pol II is regulated in trans

**Fig. 4:**
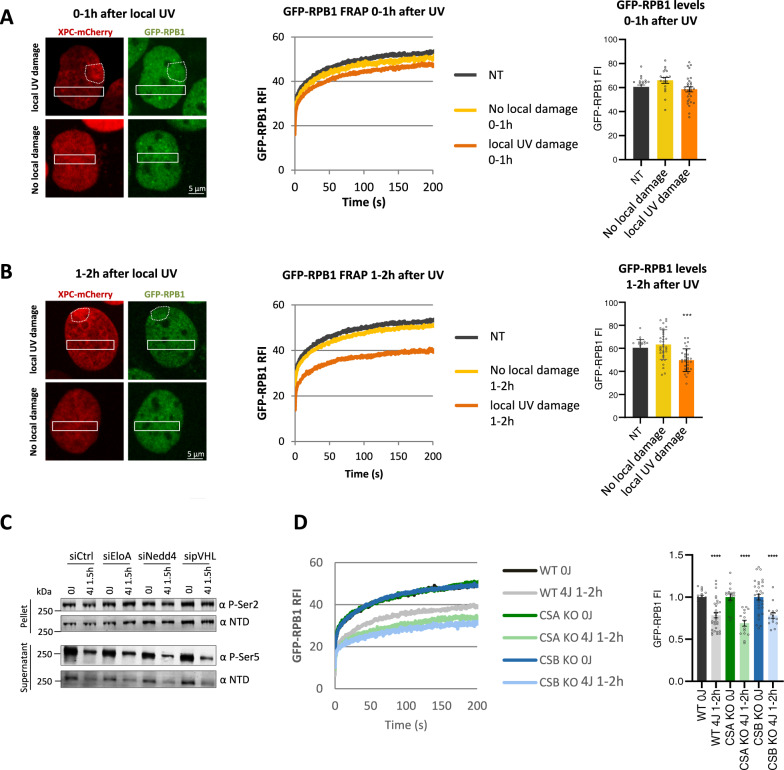
The UV-induced degradation of promoter-bound Pol II is regulated in trans. **a**, **b** Left panels: Representative image of a GFP-RPB1 KI cell expressing XPC-mCherry as a live-cell damage marker within 1 h (**a**) or 1 to 2 h (**b**) after UV irradiation through a porous filter to induce local DNA damage. Cells containing local UV-damage (top images) are marked by local accumulation of XPC-mCherry (outlined with dotted circle) or without local damage (bottom images), or non-treated cells (NT) were used for FRAP analysis. GFP-RPB1 FRAP was analyzed outside the local UV damaged, indicated by box. Middle panels: FRAP analysis of GFP-RPB1 cells that were non-treated (NT) or 0–1 h (**a**) or 1–2 h (**b**) after local UV irradiation through a porous filter. Right panels: GFP-RPB1 pre-bleach fluorescence intensity (FI) ± SD of cells analysed by FRAP as a measure for Pol II protein levels. *N* = 23 cells for ‘NT’, n = 10 cells for ‘no local damage 0–1 h’, *n* = 29 cells for ‘local UV damage 0–1 h’, *n* = 35 cells for ‘no local damage 1–2 h’, *n* = 30 cells for ‘local UV damage 1–2 h’. Cells were measured in 3 independent experiments. **c** Western blots after cellular fractionation of GFP-RPB1 knock in (KI) cells after control siRNA transfection (siCTRL), or siRNA-mediated gene knockdown of the indicated E3 ubiquitin ligases in non-perturbed conditions (0 J) or 1 h after irradiation with 4 J/m^2^ (4 J). NTD = RPB1 N-terminal domain, P-Ser = phospho-serine. Experiment has been performed two times with similar results. **d** Fluorescence recovery after photo-bleaching (FRAP) of GFP-RPB1 in wild type (WT) or in NER knock out (KO) MRC5 cells without UV irradiation (0 J) or 1 h after irradiation with 4 J/m^2^ (4 J 1 h). GFP-RPB1 was bleached and fluorescence intensity was measured every 0.4 sec, background-corrected and normalized to pre-bleach fluorescence intensity (FI), which was set to 100. RFI = relative FI. Right panel: Mean ±SD of pre-bleach GFP-RPB1 FI as a measure for Pol II protein levels in nuclei analysed by FRAP. N = 32 cells for ‘WT 4 J 1–2 h’ and ‘CSB KO 0 J 1–2 h’ measured in 4 independent experiments, *n* = 16 cells for all other conditions measured in 2 independent experiments. ***P ≤  0.001, ****P ≤  0.0001, analyzed by ordinary one-way ANOVA using Dunnett’s multiple comparisons test versus NT (**a**, **b**) or respective 0 J condition (**d**).

To investigate whether the UV-induced regulation of promoter-bound Pol II is indeed regulated in trans, we inflicted DNA damage in a sub-nuclear region by irradiation through a microporous filter^59^ and measured Pol II kinetics outside the damaged area in the same nucleus. Local UV damage was detected by co-expression of mCherry-tagged XPC. As negative controls, Pol II kinetics were measured in non-irradiated cells (NT) and in cells that were irradiated through the filter, but did not receive local UV damage (no local damage), as shown by the absence of local XPC accumulation (Fig. 4a, b left panel). Additionally, XPC was not immobilized in regions outside the local damage, as determined by FRAP of mCherry-tagged XPC, indicating that UV damage was only induced through the pores of the filter (Supplementary Fig. 3d**)**. Within the first hour after local UV irradiation, the kinetics and protein levels of Pol II transcribing undamaged chromatin in locally damaged cells (local UV damage) were similar to Pol II kinetics in control cells (Fig. 4a), indicating that the stalling of elongating Pol II *in cis* in a sub-nuclear region hardly affects Pol II kinetics in the undamaged parts of the nucleus. In contrast, 1 h after local UV irradiation, Pol II in the undamaged chromatin of locally damaged cells was markedly immobilized compared to Pol II in undamaged conditions (Fig. 4b middle panel). In addition, 1 h after UV, Pol II protein levels of locally damaged cells were reduced comparably to globally-irradiated cells (Fig. 4b and Fig. 1d, right panels). These results demonstrate that UV damage leads to the degradation of promoter-bound Pol II on non-damaged genes and that the levels of promoter-bound Pol II are regulated in trans.

The involvement of VCP in the UV-induced proteasomal degradation of promoter-bound Pol II suggests a resemblance in the degradation mechanism to that of lesion-stalled Pol II via the so-called “last resort pathway”^16^. Therefore, we tested whether the E3 ligases implicated in the degradation of lesion-stalled Pol II NEDD4^51^, ElonginA^52^, and pVHL^47^ might be involved in degradation of promoter-bound Pol II. siRNA-mediated depletion of these E3 ligases (Supplementary Fig. 3e) did not affect the UV-induced degradation of promoter-bound Pol II as shown by fractionation assays (Fig. 4c). Similarly, CRISPR-mediated gene knock out of the TC-NER factors CSA and CSB (Supplementary Fig. 5a, b**)**, which have also been reported to play a role in ubiquitylating lesion-stalled Pol II via the Cullin-RING ubiquitin E3 ligase activity of CRL4^CSA^^46,60^, did not rescue the UV-induced immobilization (Fig. 4d, left panel) or degradation (Fig. 4d, right panel) of promoter-bound Pol II. Together these results emphasize that the degradation of promoter-bound Pol II is an important component of the DNA damage-induced transcription stress response that is mechanistically distinct from the degradation of lesion-stalled, elongating Pol II.

Even though the UV-induced degradation of promoter-bound Pol II occurs independently of TC-NER, Pol II stalling at TBLs might still trigger this trans-regulatory effect. Therefore, we tested whether the degradation of promoter-bound Pol II is dependent on transcription elongation by inhibiting transcription with the CDK7 inhibitor THZ1^43^ or the CDK9 inhibitor Flavopiridol^45^ (Supplementary Fig. 1e). Transcription inhibition with either inhibitor severely decreased the levels of P-Ser2 Pol II in the pellet fraction, demonstrating efficient inhibition of transcription elongation. As expected, transcription inhibition with THZ1 greatly reduced P-Ser5 levels in the supernatant, but no additional reduction was detected after UV. Furthermore, even though Flavopiridol increased P-Ser5-modified Pol II levels in the supernatant, it completely abolished the UV-induced degradation of promoter-bound Pol II (Supplementary Fig. 1e). This indicates that productive elongation, and presumably the stalling of elongating Pol II on TBLs, is a prerequisite for the degradation of promoter-bound Pol II.

### The degradation of promoter-bound Pol II is signaled via GSK3

**Fig. 5:**
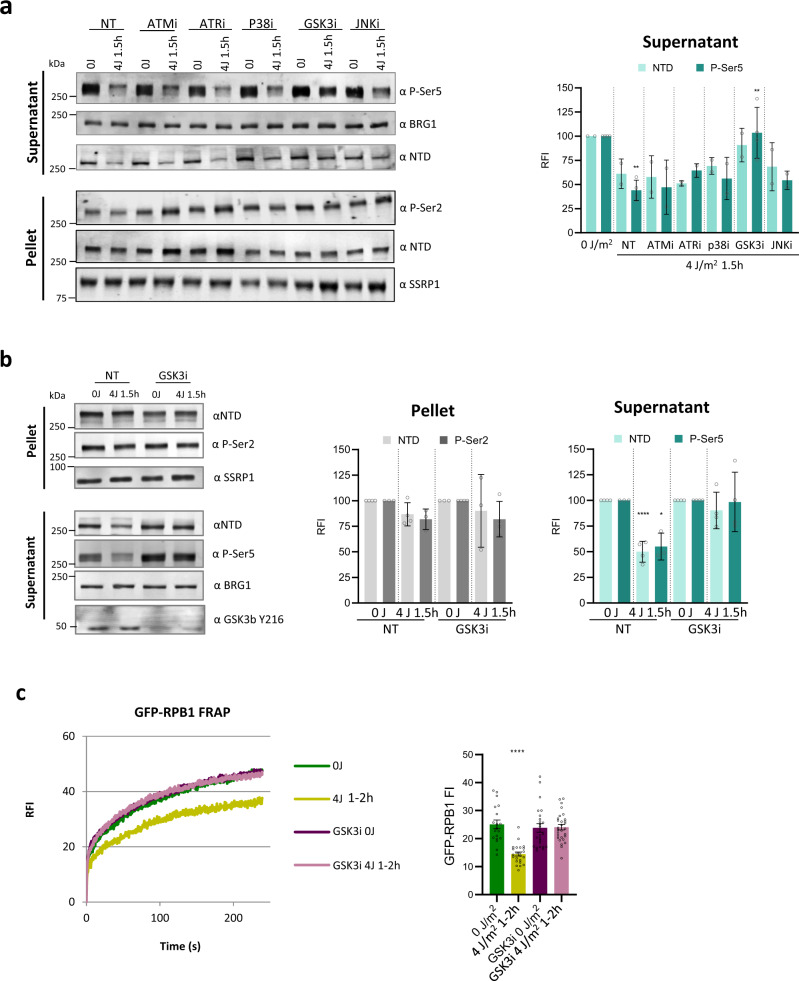
Degradation of promoter-bound Pol II is signaled via GSK3. **a** Left panel: Representative western blots after cellular fractionation of nontreated (NT) GFP-RPB1 knock in (KI) cells or cells treated with the indicated kinase inhibitors (i) 30 min before UV irradiation. Fractionation was performed in non-irradiated cells (0 J) or 1 h after irradiation with 4 J/m^2^. BRG1 was used as loading control. NTD = RPB1 N-terminal domain, P-Ser = phospho-serine. Right panel: Quantification of the western blot signals of *n* = 2 independent experiments2, mean ± SD. **b** Left panel: Representative western blots after cellular fractionation of GFP-RPB1 knock in (KI) 1 h after irradiation with 4 J/m^2^ without (NT) or with GSK3 inhibitor treatment (GSK3i, CHIR-99021, 10 μM 30 min before UV). SSRP1 and BRG1 were used as loading controls. NTD = RPB1 N-terminal domain, P-Ser= phospho-serine. Right panel: Quantification of the indicated western blot signals, mean ± SD, *n* = 3 for ‘P-Ser2’ and ‘P-Ser5’, *n* = 4 for ‘NTD’. **c** Left panel: Fluorescence Recovery after photo-bleaching (FRAP) of GFP-RPB1 after UV irradiation with or without pretreatment for 30 min with GSK3 inhibitor (CHIR-99021, 10 µM). GFP-RPB1 was bleached and fluorescence intensity was measured every 0.4 sec for 4 min, background-corrected and normalized to prebleach fluorescence intensity (FI), which was set to 100. RFI = relative FI. Right panel: Mean ±SD of pre-bleach GFP-RPB1 FI as a measure for Pol II protein levels in nuclei analysed by FRAP. *n* = 19 cells for ‘0 J’ and ‘4 J 1–2 h’, *n* = 23 cells for ‘GSK3i 0 J’, *n* = 29 cells for ‘GSK3i 4 J 1–2 h’. *P ≤  0.05, **P ≤  0.01, ****P ≤  0.0001; analyzed by ordinary one-way ANOVA using Dunnett’s multiple comparisons test versus the respective 0 J condition.

If the stalling of Pol II on TBLs is indeed the trigger to degrade promoter-bound Pol II after UV, it is tempting to speculate that the downstream regulation of this process might be propagated from lesion-stalled Pol II to promoter-bound Pol II via signaling pathways. To test this, we tested several inhibitors of kinases with established roles in the DNA damage and transcription stress responses, including ATM and ATR^61^, p38^MAPK^^62^, c-Jun N-terminal kinase (JNK)^63^, and glycogen synthase kinase 3 (GSK3)^64^. Interestingly, only the inhibition of GSK3, which was confirmed by reduced phosphorylation of Y216^65^, significantly prevented the UV-induced degradation of promoter-bound Pol II (Fig. 5a, b). In line with fractionation results, Pol II FRAP analysis after GSK3 inhibition showed that the immobilization 1 h after UV damage induction, due to degradation of promoter-bound Pol II, was absent in GSK3 inhibitor-treated cells (Fig. 5c, left panel). Furthermore, GSK3 inhibition significantly reduced the UV-induced degradation of total Pol ll levels measured by live-cell GFP-RBP1 fluorescence (Fig. 5c, right panel), while it did not affect Pol II mobility or levels directly after UV (Supplementary Fig. 4a). Similar results were obtained upon siRNA-mediated depletion of both the GSK3α and β isoforms (Supplementary Fig. 4b, c). Next, we tested whether the GSK3-mediated degradation of promoter-bound Pol II was observed only upon UV-induced damage, or could also be induced by other types of TBLs. Interestingly, also Illudin S^66^ exposure resulted in a clear degradation of P-Ser5-modified, promoter-bound Pol II, which could also be rescued by GSK3 inhibition (Supplementary Fig. 4d). Together these results not only corroborate that the degradation of promoter-bound Pol II is regulated in trans and implicate GSK3 in the signal transduction from TBL-stalled Pol II to promoter-bound Pol II, but also complement the recently described role of GSK3 in the transcriptional response to DNA damage^67^.

### Degradation of promoter-bound Pol II contributes to UV-induced transcription inhibition

**Fig. 6:**
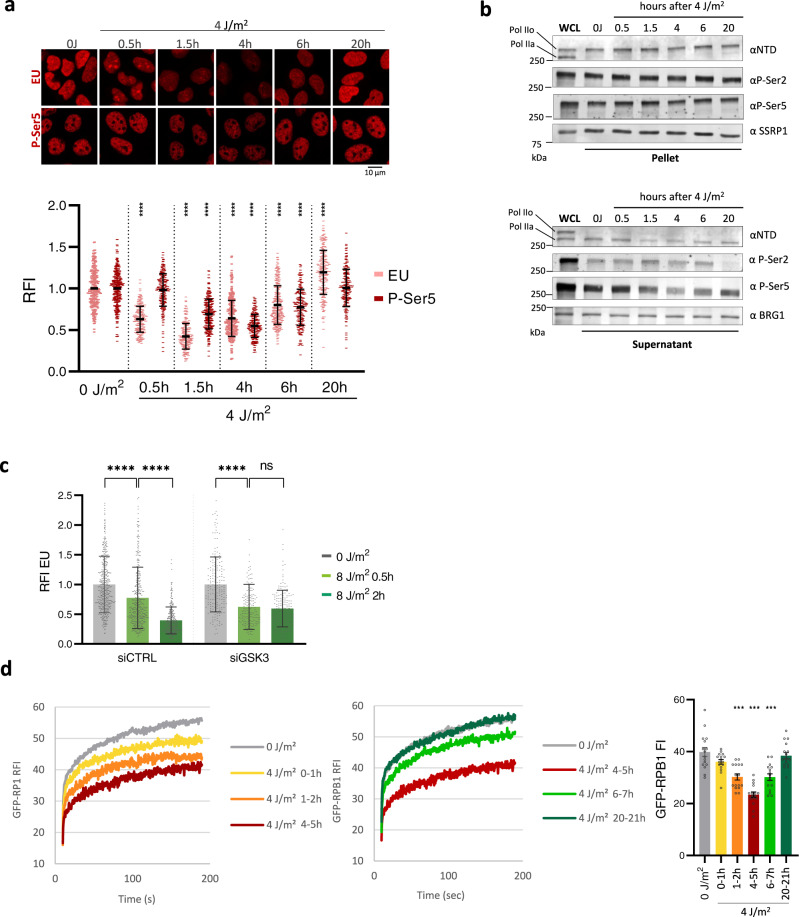
Degradation of promoter-bound Pol II contributes to UV-induced transcription inhibition. **a** Top panel: representative images of GFP-RPB1 KI cells pulse-labelled with EU for the last 30 min before fixation to visualize transcription rates by click-chemistry-based azide-594 coupling (top row), or stained with an anti-P-Ser5 RPB1 antibody (bottom row) in non-perturbed conditions (0 J) or after irradiation with 4 J/m^2^ of UV at the indicated time points. Bottom panel: EU and P-Ser5 fluorescence intensities (RFI) were normalized to 0 J/m^2^ levels, which were set to 1. *n* = 242, 329, 224, 291, 221, 301, 282, 222, 294, 202, 212, 201 cells (left to right) measured in 2 independent experiments. **b** Western blots after cellular fractionation of GFP-RPB1 knock in (KI) cells after the indicated time points after irradiation with 4 J/m^2^ of UV. Cellular fractionation was optimized to separate hyper-phosphorylated, elongating Pol IIo in the pellet from hypo-phosphorylated, free and promoter-bound Pol IIa in the supernatant. SSRP1 and BRG1 were used as loading controls. WCL = whole cell lysate, NTD = RPB1 N-terminal domain, P-Ser= phospho-serine. Experiment has been performed two times with similar results. **c** GFP-RPB1 KI cells transfected with the indicated siRNAs were pulse-labelled with EU for the last 30 min before fixation to visualize nascent transcription rates in non-perturbed conditions (0 J) or after irradiation with 8 J/m^2^ of UV at the indicated time points. EU relative fluorescence intensities (RFI) were normalized to 0 J/m^2^ levels, which were set to 1. *n* = 430, 381, 372, 186, 163, 174 cells (left to right) measured in 2 independent experiments. **d** Left and middle panel: GFP-RPB1 FRAP measurements in unperturbed cells (0 J/m^2^) or after irradiation with 4 J/m^2^. Time points indicate how long cells were left to recover before beginning FRAP measurements. GFP-RPB1 was bleached and fluorescence intensity was measured every 0.4 sec for 3 min, background-corrected and normalized to prebleach fluorescence intensity (FI), which was set to 100. RFI relative FI. Right panel: Mean ±SD of pre-bleach GFP-RPB1 FI as a measure for Pol II protein levels in nuclei analyzed by FRAP. *n* = 16 cells measured in 2 independent experiments. ***P ≤  0.001,****P ≤  0.0001; ns nonsignificant, analyzed by ordinary one-way ANOVA using Dunnett’s multiple comparisons tests.

Degradation of promoter-bound Pol II after UV irradiation is expected to cause an extra decrease in transcription levels in addition to the transcription inhibition caused by the direct stalling of Pol II at TBLs. In line with such a dual transcription inhibitory mechanism, we observed an initial drop in nascent RNA levels directly after UV (0.5 h) and an additional drop 1.5 h after UV (Fig. 6a). Since the UV-induced immobilization of elongating Pol II did not further increase between 0.5 and 1.5 h after UV irradiation (Fig. 1a, d), this suggests that the initial drop in transcription is mainly caused by the stalling of elongating Pol II on TBLs *in cis*, while the additional drop may be due to the UV-induced degradation of promoter-bound Pol II. Therefore, we tested whether this additional drop in transcription correlated with a loss of P-Ser5-modified Pol II measured by immunofluorescence (Fig. 6a) and cellular fractionation (Fig. 6b). While P-Ser5-modified Pol II levels remained unchanged within the first 0.5 h after UV, it decreased 1.5 h after UV (Fig. 6a, b). This shows that the loss of P-Ser5-modified promoter-bound Pol II correlates with the additional drop in EU incorporation. To confirm that the UV-induced degradation of promoter-bound Pol II in trans contributes to this delayed, additional transcription block, we inhibited Pol II degradation by GSK3 depletion. Upon inhibition of the GSK3-mediated degradation of promoter-bound Pol II, the additional transcription inhibition 2 h after UV was lost (Fig. 6c**and** Supplementary Fig. 4b). Together these data indicate that the degradation of promoter-bound Pol II contributes to the transcription inhibition in trans.

In line with a contribution of the degradation of promoter-bound Pol II to transcription inhibition, also the recovery of transcription after UV irradiation indicated a two-step mechanism (Fig. 6d). While Pol II remained immobilized up to 4 h after UV, 6 h after UV irradiation promoter-bound Pol II recovered greatly in mobility, as shown by upwards shift of the 6 h FRAP curve compared to the 4 h curve. In contrast, the elongation block remained persistent, illustrated by the parallel slope of the FRAP curve 6 h after UV compared to 4 h after UV (Fig. 6d). In line with the recovery of mobility of promoter-bound Pol II, also total Pol II levels (Fig. 6d, right panel) and more specifically the P-Ser5 levels (Fig. 6a, b) returned to unperturbed conditions 6 h after UV irradiation, suggesting that the transcription inhibition in trans has been suspended. Overall transcription also started to recover at 6 h post 4 J/m^2^ UV (Fig. 6a, top panel). This observation indicates that restoring the levels of promoter-bound Pol II, i.e. the recovery of transcription in trans, contributes to transcription restart independently of Pol II stalled in cis, which returned to unperturbed conditions 20 h after UV irradiation (Fig. 6a, d).

### The UV-induced regulation of promoter-bound Pol II is independent of TBL repair by TC-NER

**Fig. 7:**
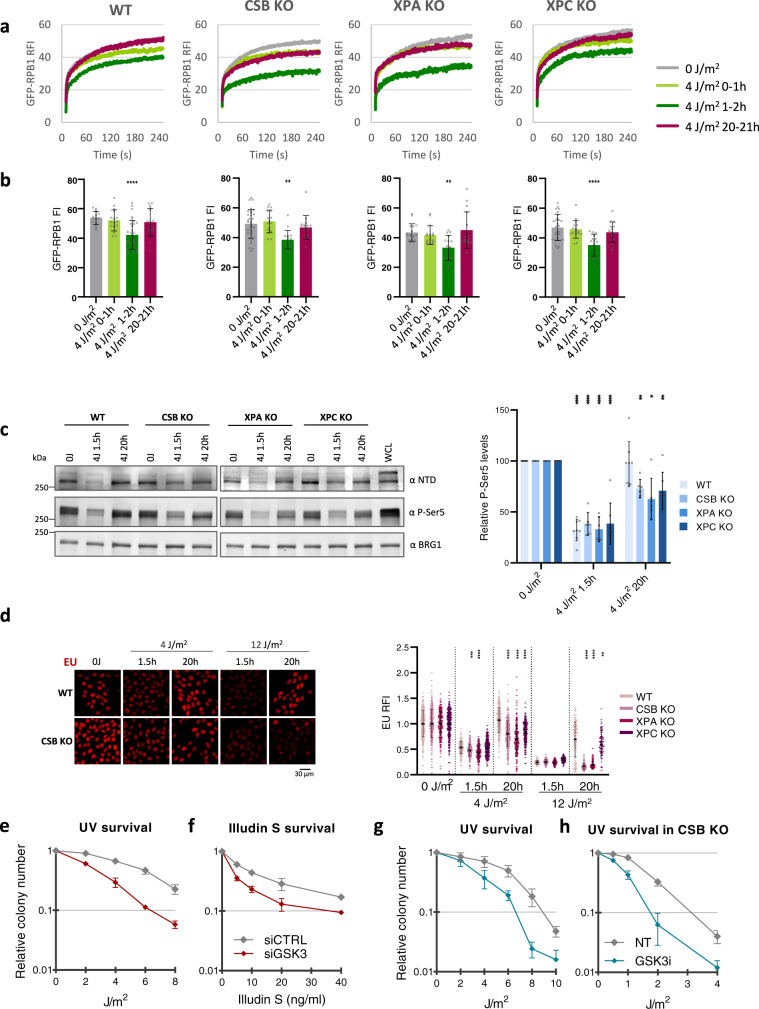
UV-induced degradation of promoter-bound Pol II is independent TC-NER and contributes to cell survival. **a** Fluorescence Recovery after photo-bleaching (FRAP) analysis of GFP-RPB1 in wild type (WT), TC-NER-deficient (CSB, XPA) cells, or in cells lacking the GG-NER damage recognizing protein XPC, in non-perturbed conditions (0 J/m^2^), immediately after irradiation (4 J/m^2^ 0–1 h), 1 h (4 J/m^2^ 1–2 h) or 20 h (4 J/m^2^ 20–21 h) after irradiation. *n* > 16 cells from 2 independent experiments. **b** Average GFP-RPB1 fluorescence intensity (FI) of KI cells analyzed by FRAP in (A) before photo-bleaching, representing Pol II protein levels at the indicated treatment conditions. *n* = 13 cells for ‘XPC KO 4 J 0–1 h’, *n* = 15 cells for ‘XPA KO 4 J 20–21 h’, *n* = 31 cells for ‘XPC KO 0 J’, *n* = 32 cells for ‘WT 4 J 1–2 h’ and ‘CSB KO 0 J’, *n* = 16 cells for all other conditions. Cells were measured in 2 independent experiments. Error bars represent SD. **c** Left panel: Representative western blots after cellular fractionation of GFP-RPB1 in wilt type cells (WT) and cells with CRISPR/Cas9-mediated gene knockout of the indicated repair factors. Fractionation was performed in nonperturbed conditions (0 J) or 1.5 h and 20 h after irradiation with 4 J/m^2^ (4 J), only the supernatant fraction containing cytoplasmic and nucleoplasmic proteins is shown. BRG1 was used as loading control. NTD = RPB1 N-terminal domain, P-Ser= phospho-serine. Right panel: Quantification of western blot band intensities of P-Ser5-modified Pol II. Mean ±SD of *n* = 6 western blot experiments for CSB and XPA KO, *n* = 7 independent western blots for XPC KO, and n = 8 independent western blots for WT. **d** Representative images (left) and quantification (right) of transcription rates in GFP-RPB1 KI cells after the indicated treatments as determined by EU pulse labelling for 30 min followed by click-chemistry based azide-594 coupling. Nuclear EU fluorescence intensity (FI) was normalized to 0 J levels, which was set to 1. *n* = 437, 397, 250, 333, 363, 428, 260, 380, 308, 354, 229, 240, 166, 196, 122, 121, 166, 166, 103, 89 cells (left to right) measured in 2 independent experiments, error bars represent SD. **e**, **f** Clonogenic survival of MRC5 wild-type GFP-RPB1 knock in (KI) cells transfected with the indicated siRNAs. Equal numbers of cells were seeded and colony forming ability was determined in triplicate 7 days after irradiation with the indicated UV doses (**e**), or 24 h exposure to the indicated dose of Illudin S. Relative colony number ± SE of 3 independent experiments is shown. **g**, **h** Clonogenic survival in either wildtype (WT) (**g**) or CSB knockout (KO) (**h**) cells upon GSK3 inhibition (GSK3i, CHIR-99021, 10 μM 30 min before UV for 24 h) upon irradiation with the indicated UV doses. Relative colony number ± SE of 3 independent experiments is shown. **P ≤  0.01, ***P ≤  0.001, ****P ≤  0.0001; analyzed by ordinary one-way ANOVA using Dunnett’s multiple comparisons tests.

As degradation of promoter-bound Pol II was independent of TC-NER (Fig. 4d), we next used TC-NER-deficient cells to distinguish the transcription restart in trans from the TC-NER-dependent transcription restart in cis^6^. Therefore, we determined Pol II kinetics after irradiation with low dose UV (4 J/m^2^) in CSB KO cells that lack the capacity to repair UV-induced TBLs by TC-NER. As control we additionally analyzed transcription recovery in GG and TC-NER deficient cells lacking the core NER protein XPA, or in cells lacking the main GG-NER damage sensor XPC (Fig. 7a). Functional knockout of repair factors was confirmed by western blot analysis (Supplementary Fig. 5a), and increased UV sensitivity (Supplementary Fig. 5b). FRAP analysis showed an increased immobilization of the long-bound fraction (>100 sec) within the first hour after UV, followed by the additional loss of promoter-bound Pol II one hour after UV in all cell types. In line with the absence of TBL repair, in TC-NER deficient cells (CSB and XPA KO) the immobilization of Pol II was stronger than in WT and XPC cells, which was most evident 1 h after UV damage. As expected, WT and GG-NER deficient XPC KO cells featured a full transcription recovery 20 h after UV. Strikingly, also TC-NER-deficient cells showed a significant recovery of Pol II kinetics 20 h after TBL infliction with 4 J/m^2^, however, the TBL-induced immobilization of the long-bound fraction (>100 sec in the FRAP curves), representing lesion-stalled elongating Pol II, persisted in TC-NER-deficient cells. Moreover, GFP-RPB1 fluorescence levels (Fig. 7b) and P-Ser5 levels (Fig. 7c) recovered significantly in all cell lines. These data indicate that the levels of promoter-bound Pol II in trans recovered after low dose UV in TC-NER-deficient cells, while the stalling of Pol II at TBLs in cis did not. To confirm this, we determined transcription levels by EU incorporation in TC-NER-deficient cells 20 h after UV-irradiation with 4 J/m^2^. Strikingly, we observed a partial recovery of transcription in TC-NER-deficient CSB and XPA KO cells, whereas TC-NER-proficient WT and XPC KO cells recovered fully (Fig. 7d). This finding was highly unexpected, as the restart of transcription after UV damage was thus far fully attributed to TC-NER activity. Our results however show, that at low DNA damage loads, even in the presence of unrepaired TBLs on some genes, the recovery of promoter-bound Pol II allows a partial, but significant resumption of transcription, presumably on undamaged genes. In line with such a model, after irradiation with a damage load that inflicts TBLs in almost all genes (12 J/m^2^), promoter-bound Pol II kinetics (Supplementary Fig. 5c), total Pol II levels (Supplementary Fig. 5d), P-Ser5-modified Pol II in the supernatant fraction (Supplementary Fig. 5e), and transcription levels (Fig. 7d) only recovered in TC-NER proficient, but not in TC-NER deficient cells.

To study the biological relevance of this uncovered layer of regulation in the intricate cellular response to DNA damage-induced transcription stress, we inhibited the DNA damage-induced degradation of promoter-bound Pol II by siRNA-mediated depletion of the GSK3α and β isoforms (Supplementary Fig. 4b). GSK3 depletion resulted in a hypersensitivity to UV- and Illudin S-induced DNA damage (Fig. 7e, f), indicating that GSK3 plays an important role in the cellular protection against TBLs. Similar results were obtained by GSK3 inhibition (Fig. 7g). In line with the observation that degradation of promoter-bound Pol II is independent of TC-NER and contributes to transcription inhibition and restart in TC-NER-deficient cells, GSK3 inhibition further sensitized CSB knock-out cells to UV damage. In conclusion, our results show that the recovery of promoter-bound Pol II contributes to the restart of transcription after UV and confirms that the degradation of promoter-bound Pol II significantly contributes to the transcription inhibition after UV, which is an important layer of regulation of the cellular protection to DNA damage-induced transcription stress.

## Discussion

In this study we discerned two independent mechanisms that contribute to TBL-induced transcription inhibition and the subsequent transcription restart: stalling of Pol II on TBLs in cis, and the VCP-mediated genome-wide degradation of promoter-bound Pol II in trans. We show that this trans-regulatory effect is uncoupled from DNA repair by TC-NER and is therefore mechanistically separated from the processing of lesion-stalled Pol II, and represents an additional mechanism in the cellular response to DNA damage-induced transcription stress. In addition, our findings disclose a yet undescribed role for the GSK3 kinase and the VCP segregase in the UV-DDR: the genome-wide extraction of ubiquitylated, promoter-bound Pol II from chromatin in response to TBLs.

Our observations that promoter-bound, P-Ser5-modified Pol II is targeted for degradation suggests that promoter-proximal paused Pol II is the main Pol II form that is degraded in trans, as this Pol II form is characterized with a P-Ser5 modification without a P-Ser2 modification^2^. In line with such a model, Pol II ChIP experiments showed that the Pol II peak at the transcription start site (TSS), which mainly represents promoter-paused Pol II^2^, is lost upon UV in a VCP-dependent manner (Fig. 3a, c**and** Supplementary Fig. 3b). However, as initiating and promoter-paused Pol II continuously cycle through the stages of the transcription cycle, affecting one fraction will most likely directly affect the other. While we cannot exclude that also initiating Pol II is regulated by degradation in trans, the fact that upon UV damage P-Ser5-modified Pol II was ubiquitylated (Fig. 3b), and P-Ser5-modified Pol II was stabilized upon VCP and proteasome inhibition (Fig. 2), shows that Pol II can reach the promoter-paused stage before being targeted for degradation. Moreover, taking into the account the highly transient nature of initiating Pol II with an average binding time of 2–6 s^33,68^, promoter-paused Pol II seems the more likely candidate for this ubiquitin-mediated regulation of promoter-bound Pol II.

### Lesion-stalled and promoter-bound Pol II is processed independently after UV irradiation

Using a recently developed sensitive live cell-imaging approach to analyze Pol II kinetics^33^ allowed us to distinguish the stalling of elongating Pol II on TBLs *in cis* from the effects of UV on promoter-bound Pol II in trans at physiologically relevant damage loads of 4 J/m^2^ UV, which leaves most genes undamaged^36^. Direct effects of TBLs on elongating Pol II *in cis* became apparent by the dose-dependent increase of Pol II immobilization immediately (0–1 h) after TBL infliction (Fig. 1a). Monte-Carlo-based modeling of Pol II mobility^33^ indicated that this immobilization is caused by a 25% increase in residence time of elongating Pol II after TBL induction (Fig. 1b, c). This is indicative for a reduced Pol II elongation rate and is most likely explained by the stalling of a subset of Pol II at TBLs awaiting repair by TC-NER. In line with such a scenario, in TC-NER-deficient cells elongating Pol II was more severely immobilized (Fig. 7a). Nevertheless, our data do not rule out that a small subset of lesion-stalled Pol II might be released from chromatin as previously suggested^15^. However, Pol II FRAP analysis revealed a significant immobilization of Pol II up to 6 h after UV irradiation (Fig. 6d), suggesting that the majority of lesion-stalled Pol II remains chromatin-bound.

In contrast to the direct stalling of Pol II at TBLs, the degradation of promoter-bound Pol II was most pronounced 1 hour after UV irradiation (Fig. 1d), and was equally prominent at low and high damage loads (Fig. 1f, g). These contrasting kinetics suggests a functional distinction of the two events and support a signal-transduced regulation of the observed effects in trans. Whereas the stalling of Pol II at TBLs initiates TC-NER^6^, the delayed response in trans resulted in the degradation of non-lesion stalled Pol II at the promoter, and occurred independently of TC-NER. This corroborates that the processing of TBL-stalled Pol II in cis and the degradation of promoter-bound Pol II in trans are individually regulated components of the transcription stress response. Moreover, the ubiquitin E3 ligases Elongin A, Nedd4 and pVHL, which were previously described to be involved in ubiquitylation and degradation of lesion-stalled Pol II^46,47,51,52^, were not implicated in the degradation of promoter-bound Pol II (Fig. 4c, d). This finding emphasizes that also the molecular mechanisms to process Pol II in cis and in trans upon transcription stress are distinct.

### The degradation of promoter-bound Pol II is regulated in trans

The analysis of Pol II promoter occupancy by ChIP-Seq after VCP inhibition and low dose UV irradiation (Supplementary Fig. 3b) revealed that VCP mediates the extraction of paused Pol II on virtually all promoters, although after 4 J/m^2^ only a fraction of genes is expected to be damaged^36^. Together with the observation that locally-damaged DNA induced the loss of promoter-bound Pol II on non-damaged DNA within the same cell (Fig. 4b), this alludes to a signaling-activated degradation of Pol II in a genome-wide manner. In line, we found that the degradation of promoter-bound Pol II is dependent on active GSK3 signaling (Fig. 5). Besides its early-identified role in metabolism, GSK3 has a large number of additional phosphorylation targets involved in different signaling pathways, and thereby regulates a variety of biological processes, including gene expression, cell survival, and cell proliferation^64,69^. In line with a role for GSK3 in the transcription stress response, it was recently identified to phosphorylate Pol II upon DNA damage and thereby regulating alternative splicing^67^. While it is tempting to speculate that the GSK3-mediated phosphorylation of RPB1 itself could result in a phosphodegron-based degradation of Pol II, we could not observe any effect for βTRCP or FBXW7 (data not shown), proteins that can target GSK3-phosphorylated proteins for degradation^70,71^. The exact GSK3 target and the downstream pathway that regulates the UV-induced degradation of promoter-bound Pol II still need to be unraveled, however, its involvement in the process confirms that the degradation of promoter-bound Pol II is signaled in trans.

The initial trigger that activates the signaling for the observed effect in trans effect remains unknown. We have excluded the involvement of damage recognition by TC-NER or GG-NER and NER-mediated damage processing, using CSB, XPC and XPA-deficient cells, respectively (Figs. 4d and 7a). However, we found that the degradation of promoter-bound Pol II depends on productive Pol II elongation (Supplementary Fig. 1e), suggesting that the stalling of Pol II on TBLs in cis might trigger Pol II regulation in trans. Furthermore, the degradation is not specific to UV-induced DNA damage, as a similar response was observed for the TBL-inducing agent Illudin S^66^ (Supplementary Fig. 4d).

In contrast to the genome-wide degradation of promoter-bound Pol II 1 h after local damage infliction (Fig. 4b), no elongation block was observed in the non-damaged chromatin (Fig. 4a, b at time points > 100 sec). This suggests that under these conditions, the stalling of Pol II *in cis* does not elicit a signaling response to elongating Pol II on non-damaged genes, and therefore suggests that TBL-induced reduction in elongation speed is mainly caused by the direct stalling of Pol II on a TBL^9,30,72^.

### An additional role for VCP in the transcription stress response

The importance of the ubiquitin-specific segregating function of VCP has been described for multiple DNA repair factors^73^. However, its role in the DNA damage-induced response to transcription stress has thus far been attributed to the extraction of lesion-stalled Pol II, under conditions where TC-NER becomes limiting^16,56^. Our results suggest that VCP has an additional function to extract promoter-bound Pol II. At the low damage loads used here, we were unable to detect significant effects of VCP inhibition on elongating Pol II (Fig. 2b, c), and we did not observe severe degradation of elongating Pol II after irradiation with 4 J/m^2^ (Fig. 1g, h). Furthermore, VCP inhibition rescued specifically the P-Ser5-modified form of Pol II after UV (Fig. 2c), which indicates that the depletion of promoter-bound Pol II is not caused by inhibited Pol II initiation^22–24^. Interestingly, upon VCP or proteasome inhibition, an increase of P-Ser5-modified Pol II was observed, suggesting that degradation of promoter-bound Pol II may also play a role in its turnover in undamaged conditions^74,75^. Together these data show that when damage loads are low and TC-NER is active, the most prominent function of VCP is to extract promoter-bound Pol II, and not elongating Pol II, from chromatin to allow its proteasomal degradation.

### The biological relevance of degrading promoter-bound Pol II

The genome-wide shut-down of transcription by degradation of promoter-bound Pol II seems a fairly drastic response to low damage loads in cells that are equipped with efficient DNA repair mechanisms, but it is in line with previously reported mechanisms to inhibit transcription after UV^22–24^, e.g. by sequestration of TFIIH from transcription start sites to repair complexes, eventually leading to reduced promoter binding of Pol II^22^. However, in contrast to the TC-NER-independent degradation of promoter-bound Pol II described here, this process was CSB-dependent^22^ and did not affect Pol II protein levels, strongly suggesting that these events represent distinct processes.

An interesting question is what the biological significance of degrading promoter-bound Pol II might be, and why it occurs one hour after the initial Pol II stalling *in cis*. Recently, damage-induced Pol II promoter-proximal pause release^27^ was reported, e.g. by RBM7-mediated PTEF-b activation^26^, or through P38^MAPK^-activated chromatin release of NELF^76^. Importantly, these effects are activated immediately upon stress induction (0–30 min) and are described to accelerate TBL sensing by elongating Pol II and to provide a stress-adapted transcriptome. In contrast, the genome-wide degradation of promoter-bound Pol II described here only became apparent after a significant time delay (>60 min) compared to the initial stalling of elongating Pol II (Figs. 1, 3a and 4a, b). Furthermore, stress-induced PTEF-b activation is dependent on Ca2^+^ signaling^77^, inhibition of which did not prevent the UV-induced degradation of promoter-bound Pol II (Supplementary Fig. 6). Taken together this clearly separates the two effects and implies distinct temporarily-separated biological functions.

While the early response of unleashing Pol II may aid quick TBL recognition and activation of TC-NER, the delayed degradation of promoter-bound Pol II might serve to terminate these early responses by shutting down transcription genome-wide. This would provide time for the TC-NER machinery to remove TBLs without new Pol II being unleashed into damaged genes, thereby avoiding further catastrophe provoked by lesion-stalled Pol II. This delay might be caused by the time needed to sense TBLs, for example on long or slowly transcribed genes. Such a postponed transcription inhibition might warrant synthesis of essential mRNA transcripts needed to sustain an efficient cellular response to transcription stress directly after a genotoxic insult^26,31,76^. After this immediate stress response is complete, degrading promoter-bound Pol II might reduce the formation of DNA damage-induced R-loops^78^, or may avoid highly cytotoxic collisions between replication machineries and lesion-stalled Pol II^17^, thereby safeguarding genomic integrity and cellular function^5^. In addition, degrading promoter-bound Pol II would terminate Pol II release in putatively damaged gene bodies, which might allow alternative, non-transcription-coupled repair pathways such as GG-NER to remove TBLs^6,72^. As GSK3 inhibition resulted in a severe sensitivity to TBLs, this suggests that the degradation of promoter-bound Pol II helps cells to cope with the detrimental effects of DNA damage-induced transcription stress.

The Pol II promoter-proximal pause site is recognized as an important integrative hub that allows quick adaption of transcriptional programs to cellular needs upon changes in the environment^79^. Our data, together with recent studies^26,27,76^, shows that promoter-proximally paused Pol II is not only important to adapt transcription in unperturbed condition, but also plays an essential role in the transcriptional responses to DNA damage. Based on our findings, the recently evolving model that the cellular response to transcription stress comprises a trans-regulatory component can be expanded to include the genome-wide degradation of promoter-bound Pol II as an additional, DNA-repair independent mechanism to down-regulate transcription in trans in response to TBLs.

## Methods

For information about inhibitors, antibodies, siRNAs and sgRNAs see Supplementary Table 1.

### Cell culture

All experiments were performed in homozygous GFP-RPB1 knock-in MRC-5 human lung fibroblasts (sv40-immortalized)^33^. Cells were cultured in a 1:1 mixture of Ham’s F10 and DMEM (Gibco), supplemented with antibiotics (penicillin, streptomycin) and 10% fetal calf serum, at 37˚C; 20% O_2_, and 5% CO_2_ in a humidified incubator. For fractionation experiments with Illudin S, cells were treated with 0.1ug/ml illudin S for 75 min prior harvest. GSK3 inhibitor (GSK3i, CHIR-99021, 10 μM) was added 1 h before illudin S treatment.

### UV irradiation

Cells were washed with PBS and after PBS removal exposed to UV-C light emitted by a 254 nm germicidal lamp (Philips). Local UV damage was inflicted through an isopore membrane filter (Millipore) with a pore size of 5 µm^59^.

### siRNA transfection

Cells were transfected with the indicated siRNAs (150 pmol) using RNAiMax (Thermo Fisher Scientific) 48–72 h prior to the experiment, according to manufacturer’s protocol. The siRNAs were purchased from Dharmacon and sequence information can be found in Supplementary Table 1.

### RNA isolation, cDNA synthesis and RT-qPCR

To determine ELO-A, NEDD4, and VHL expression levels, RNA was isolated using the RNeasy mini kit (Qiagen) and cDNA was synthesized using SuperScript™ II Reverse Transcriptase (Invitrogen), both according to the manufacturer’s protocol. The generated cDNA was amplified by standard RT-qPCR using SYBR green and run on a CFX96 Touch™ Real-Time PCR Detection System (Biorad). PowerUp SYBR green master mix (Thermo Fisher) was used according to manufacturer’s protocol. Samples were loaded in triplicate and the following program was used: 50 °C for 2 min., 95 °C for 2 min., 45 cycles of 15 sec. at 95 °C and 1 min. at 58 °C followed by a dissociation curve: 95 °C for 10 sec. and heating from 65 °C to 95 °C with an increment of 0.5 °C, 5 sec. each. Data collection was enabled at each increment of the dissociation curve. mRNA expression levels were normalized to GAPDH using the 2-ΔΔCt method.

### Generation of cell lines

Repair-deficient GFP-RPB1 KI cell lines were generated by targeting the RPB1 locus with GFP as described^33^ in single-cell knock-out clones for CSA, CSB, XPA and XPC that were previously generated using either the lentiCRISPR v2 vector^80^ (CSA, CSB) or a dual, doxycycline-inducible CRISPR/Cas9 vector system (iKRUNC)^81^ (XPA, XPC).

### Clonogenic Survival

Cells were seeded in triplicate in 6-well plates (300 cells/well) and treated with indicated UV doses by a single, timed exposure to ta 254 nm TUV lamp (Philips) 1 day after seeding. Illudin S treatments were also conducted 1 day after seeding and cells were washed with PBS 24hrs after exposure to illudin S. After 1 week, colonies were fixed and stained in 50% methanol, 7% acetic acid and 0.1% Coomassie blue and subsequently counted with the Gelcount (Oxford Optronix, Software Version 1.1.2.0). The survival was plotted as the mean percentage of colonies detected following the indicated UV dose compared to the mean number of colonies from the non-irradiated samples.

### Cell fractionation and Western Blotting

Cells were grown to 80% confluence in 6 cm dishes. After indicated treatments whole-cell lysates were prepared by sraping the cells in 150 µl lysis buffer (30 mM Hepes pH7.6, 1 mM MgCl_2_, 130 mM NaCl, 0.5% Triton, 50 µM Mg132, EDTA-free protease inhibitor (Roche), and phosphatase inhibitor cocktail 2 (Sigma Aldrich)). 500U of benzonase (Millipore) was added per sample and incubated for 1 h on ice. For chromatin fractionation whole cell lysates were centrifuged for 15 min at 16100 × g and 4 °C. The supernatant containing nucleoplasmic RPB1 was collected. The pellet containing chromatin-bound RPB1 was washed twice with lysis buffer and re-suspended in 150 µl lysis buffer. Supernatant and pellet were diluted with 150 µl 2x SDS Page loading buffer (4% SDS, 0.2% bromophenol blue, 20% glycerol, 200 mM β-mercaptoethanol) and separated on a 6% SDS PAGE gel. Proteins were transferred overnight at 4 °C at 35 V in 2x transfer buffer (25 mM TRIS, 190 mM Glycine) without methanol. Immobilon P PVDF membranes (Carl Roth) were blocked with 1.5% BSA in PBS and stained with antibodies as listed in Supplementary Table 1. Secondary antibodies were coupled to IRDyes (LiCor) and imaged with an Odyssey CLx infrared scanner (LiCor).

### Isolation of ubiquitylated proteins

Ubiquitylated proteins were isolated from the supernatant fractions after cellular fractionation as described above using Tandem Ubiquitin Binding Entities (TUBE2) coupled to agarose beads^58^(Boston BioChem) according to manufacturer’s instructions. Per condition 30 million cells were lysed 1.5 ml lysis buffer and fractionated. To elute and denature ubiquitylated proteins, beads were boiled in 2x SDS PAGE loading buffer (4% SDS, 0.2% bromophenol blue, 20% glycerol, 200 mM β-mercaptoethanol) for 3 min. Ubiquitylated proteins were separated by SDS PAGE as described for western blotting. Information about antibodies can be found in Supplementary Table 1.

### Immunofluorescence

Cells were grown to 80% confluency on glass coverslips, treated as indicated, fixed with 2% PFA in PBS for 15 min at room temperature. After permeabilisation with 0.1% Triton in PBS for 10 min and blocking in 1.5% BSA and 0.15% glycine in PBS for 10 min cells were incubated with primary antibodies diluted in blocking buffer for 2 h at RT. Antibodies and respective dilutions are listed in Supplementary Table 1. After washing in 0.1% triton in PBS for 10 min cells were incubated with respective secondary antibodies coupled to the indicated Alexa fluorophores in blocking buffer for 2 h at RT. Coverslips were washed with PBS containing 0.1% Triton and mounted with Vectashield containing DAPI (Brunschwig Chemie). Cells were imaged with a Zeiss LSM 700 Axio Imager Z2 upright microscope equipped with a 63x Plan-Apochromat oil immersion lens (NA 1.40). Average, nuclear fluorescence intensities were quantified after defining cell nuclei by DAPI staining using the particle analysis tool of the Fiji software^82^. The mean fluorescence intensity plotted represents the average of the relative nuclear fluorescence ± standard error of the mean of all cells measured across the independent experiments. For high content microscopy cells were seeded in UV-Star® μClear® 96 well black microplates (Greiner Bio Science). For UV irradiation plates were covered with adhesive PCR plate foils (Thermo Fisher), turned upside down, and irradiated through the UV-permeable cycloolefin bottom film. Image acquisition was performed using a spinning disk confocal Opera Phwaenix™ HCS system (Perkin Elmer) equipped with a 40x water immersion objective (NA 1.1) and 405, 488, and 561 nm solid-state lasers. GFP and P-Ser signals was detected using a 500–550 and 570–630 nm band pass filter, respectively. Images were analyzed using the Harmony® High Content Imaging and Analysis Software (Perkin Elmer).

### EU incorporation

Transcription levels were measured by pulse labeling with the nucleotide analogue 5′ethynyl uridine (EU) (Jena Bioscience). Cells were grown to 80% confluency on glass coverslips, treated as indicated, and incubated for 30 min with 1 µM EU in Ham’s F10 medium supplemented with 10% dialyzed fetal calf serum (Gibco). Subsequently, cells were washed with PBS, fixed with 2% PFA in PBS for 15 min. After permeabilisation with 0.1% triton in PBS for 10 min, click chemistry-based azide coupling was performed by incubation for 30 min with 60 µM Atto594 Azide (Attotec, Germany) in 50 mM Tris buffer (pH 8) with 4 mM CuSO_4_ (Sigma Aldrich) and 10 mM freshly prepared ascorbic acid. Coverslips were washed with PBS and mounted with Vectashield containing DAPI (Brunschwig Chemie). All steps were performed at RT. Cells were imaged with a Zeiss LSM 700 Axio Imager Z2 upright microscope equipped with a 63x Plan-Apochromat oil immersion lens (NA 1.40) using Carl Zeiss LSM (version 14.0.0.0) and image analysis was performed using Image J.

### Live cell imaging and FRAP

Live cell imaging was performed on a Leica SP5 confocal laser scanning microscope with a HCX PL APO CS 63x, 1.40NA oil immersion lens. GFP was excited with a 488 nm Argon laser and emission was recorded between 500 and 600 nm. Data was analyzed using Leica LAS AF (version 2.7.4.10100) and LAS X (version 3.5.6.21594) software. Cells were seeded on glass coverslips two days prior to the experiment so that they would reach full confluency on the day of the experiment. For FRAP, at zoom 10, a strip of 512 × 32 pixels spanning the nucleus was imaged every 400 ms with 400 Hz. 25 frames were recorded before the bleach pulse. GFP fluorescence in the strip was bleached for 1 frame with 100% laser power. The recovery of fluorescence was monitored for 3–4 min within and outside the strip. Fluorescence intensity within the strip was background-corrected for the fluorescence intensity outside the strip. The average, background-corrected fluorescence intensity of frame 10 to 20 of pre-bleach measurements was used to calculate the pre-bleach fluorescence intensity. The background-corrected pre-bleach fluorescence intensity was set to 100% and post-bleach measurements were normalized to pre-bleach measurements. The background-corrected and normalized fluorescence recovery of 8–10 cells was averaged per experiment. FRAP curves show the average of all cells measured in 2–4 experiments, as indicated in figure legends.

To study in trans effects of UV-induced DNA damage, we generated stable XPC-mCherry expression in the MRC-5 GFP-RPB1 KI cells. Local UV damage was induced in these cells by 40 J/m^2^ UV-C through an 8 μm microporous filter^59^. RPB1 mobility in cells with a local damage, as determined by a local accumulation of XPC-mCherry, was determined is a strip outside the damage region. As control, RPB1 mobility was also observed in cells irradiated but without a local damage, these cells did not receive detectable UV damage as they were shielded by the filter.

### Chromatin immunoprecipitation combined with RT-qPCR analysis

One full 10cm^2^ dish of MRC-5 wild-type or knock in cells was used per condition. Following inhibitor treatment, chromatin was cross-linked using 1% formaldehyde in culture medium for 10 min. Crosslinking was quenched by adding glycine to a final concentration of 0.125 M for 5 min. Cells were washed twice with PBS and scraped in PBS before pelleting by centrifugation. Pellets were lysed for 10 min. in sonication buffer (0.1% SDS, 10 mM Tris-HCL pH8, 1 mM EDTA, 0.5 mM EGTA) including complete protease inhibitor cocktail without EDTA (Roche), phosphatase inhibitor cocktail 2 and 3 (Sigma). Samples were sonicated for 15 min using Bioruptor (Diagenode, 15 sec on, 15 sec off, amplitude High) to fragments of 200–500 base pairs. Chromatin was diluted using dilution buffer (0.01% SDS, 1.1% Triton X-100, 1.2 mM EDTA, 16.7 mM Tris-HCL pH8, 167 mM NaCl) and pre-cleared for 30 min using Pierce Protein G Agarose beads (Thermo Scientific). RPB1 was immuno-precipitated overnight at 4 °C using 5 µg GFP antibody (5 mg/ml, ab290, Abcam) and for an additional hour with Pierce Protein G Magnetic beads (Thermo Fisher). Precipitates were washed for 3 min with low salt buffer (0.1% SDS, 1% Triton X-100, 2 mM EDTA, 20 mM Tris-HCL pH8, 150 mM NaCl), high salt buffer (0.1% SDS, 1% Triton X-100, 2 mM EDTA, 20 mM Tris-HCL pH8, 500 mM NaCl), lithium chloride buffer (0.25 M LiCl, 1% NP-40, 1% Sodium Deoxychelate, 1 mM EDTA, 10 mM Tris-HCL pH8) and twice with Tris-EDTA buffer (10 mM Tris-HCL pH8, 1 mM EDTA). DNA was eluted from beads twice using elution buffer (1% SDS, 0.1 M NaHCO_3_) and crosslink was reversed for 4 h with 0.2 M NaCl at 65 °C and 950 rpm in a thermoshaker. Proteins were degraded by incubating 1 h at 45 °C and 700 rpm with Proteinase K, Tris-HCL pH 6.5 and EDTA before cleaning the DNA using a DNA ChIP Clean & Concentrator kit (Zymo Research). qPCR was performed using the CFX96 Touch™ Real-Time PCR Detection System (Biorad) and PowerUp SYBR green master mix (Thermo Fisher) according to manufacturer’s protocol. Primer sequences as found in Supplementary Table 1 were used to amplify genomic DNA of RPLP1, RPL3 and DYNLL1. Primer sequences were shown to linearly amplify the expected genomic regions. Samples were loaded in duplicate and the following program was used: 50 °C for 2 min, 95 °C for 2 min, 45 cycles of 15 sec at 95 °C and 1 min at 58 °C followed by a dissociation curve: 95 °C for 10 sec, 65 °C for 5 sec and heating from 5 °C to 95 °C. Enrichment of bound DNA was calculated as percentage of the input and normalized for the NT sample.

### Chromatin immunoprecipitation combined with sequencing

Chromatin was extracted and sheared as described^83^. Briefly, cells were fixed in 1% methanol-free formaldehyde (Thermo Scientific, 28906) in PBS at room temperature for 10 min, followed by 5 min blocking in 0.125 M glycine. Cells were washed twice with ice-cold PBS. The cell pellet was resuspended in Farnham buffer (5 mM PIPES, pH 8; 85 mM KCl; 0.5% Igepal). Cell suspensions were sonicated for 4 min in 1 ml Covaris tubes (Covaris, 520130) using Covaris S220 with the following settings: peak power = 75; duty factor = 2; cycles/burst = 200. Isolated nuclei were washed with Farnham buffer and suspended in shearing buffer (10 mM Tris-HCl, pH 8; 0.1% SDS; 1 mM EDTA). Chromatin was sheared by sonication for 12 min in 1-ml Covaris tubes using the following settings: peak power = 140; duty factor = 5; cycles/burst = 200. Debris was removed by centrifugation. A DNA fragment–size distribution of 200–600 bp was considered as ideal chromatin for ChIP. Chromatin was diluted 1:1 with IP buffer (10 mM Tris-HCl, pH 8; 100 mM NaCl, 1 mM EDTA, 0.5 mM EGTA, 0.1% sodium deoxicholate, 0.1% *N*-lauroylsarcosine) to achieve a final 0.05% SDS concentration. Good quality chromatin (200 μg) was used for immunoprecipitation as described^84^. Briefly, Protein A magnetic beads (Life Technologies, 10002D) were incubated (rotated) with 10 μg of f Rpb1 NTD (D8L4Y) Rabbit mAb (Cell Signaling #14958) for 6 h at 4 °C. This bead–antibody complex was then incubated overnight at 4 °C with chromatin. As normalization method for RNA Pol II ChIP-Seq, mouse chromatin was used according to the spike-in method described^85^. Briefly, mouse chromatin from embryonic stem cells was independently crosslinked and sheared to obtain the same size distribution as in human chromatin used in all experiments, and then aliquoted and stored. When required, this control chromatin was mixed with chromatin from treated human cells at a 2.5:97.5 ratio and immunoprecipitation was performed. An aliquot of chromatin (containing mouse spike-in chromatin) was saved as input DNA. Beads were washed and DNA–protein complexes were eluted from the beads by heating at 65 °C in elution buffer (50 mM Tris-HCl, pH 8.0, 10 mM EDTA and 1% SDS). Crosslinking was reversed for 6 h at 70 °C and samples were treated with 200 μg ml^–1^ RNase A (Applichem, A3832) and 200 μg ml^–1^ proteinase K (Sigma-Aldrich, P2308). ChIP DNA was purified with phenol–chloroform extraction and ethanol precipitation and used either for ChIP-qPCR or library preparation for next-generation sequencing. For ChIP-qPCR, enrichment of the immunoprecipitated DNA at the corresponding loci was expressed as a percentage relative to the input DNA. For ChIP-seq, DNA libraries were prepared from immunoprecipitated DNA. Sequencing libraries were prepared using the NEBNext Ultra II DNA Library Prep kit for Illumina (NEB E7645S). Some 2–5 ng of immunoprecipitated DNA was used for library preparation. Library size distribution was monitored by capillary electrophoresis (Agilent 2100 Bioanalyzer, High Sensitivity DNA Chips (Agilent, 5067–4626)). Libraries were sequenced paired-end on HiSeq 2500, HiSeq 3000 or NextSeq 500 instruments (Illumina). For each experiment, at least two biological replicates were generated and sequenced with a minimum depth of 40 million for RNA Pol II immunoprecipitation. Sequencing analysis was done as described^86^.

### Monte Carlo-based modelling

Simulation of GFP-RPB1 FRAP measurements was performed as described previously^33^, with the modification that the short-and medium Pol II fractions were combined as one promoter-bound fraction during modelling.

### Statistical analysis

Statistical analyses were performed using Graph Pad Prism version 9 for Windows (GraphPad Software, La Jolla California USA). *P* values expressed as *P  <  0.05; **P  <  0.01, ***P  <  0.001, **** *P*  <  0.0001 were considered to be significant. ns, non-significant. Ordinary one-way ANOVA and Dunnett’s multiple comparisons test was used to determine significance between groups of individual data points. In all experiments, between-group variances were similar and data were symmetrically distributed. Mean values ± standard deviation (SD) or standard error of the mean (SE) were plotted as indicated.

### Reporting summary

Further information on research design is available in the Nature Research Reporting Summary linked to this article.

## Supplementary information

Supplementary Information

Reporting Summary

## Data Availability

The data that support this study are available from the corresponding author upon reasonable request. All Pol II ChIP-seq data that support the findings of this study have been deposited in NCBI’s Gene Expression Omnibus and are accessible through GEO Series accession number GSE169480. Source data are provided with this paper.

## Source data

Source Data
