# Supplementary material for: DNA damage-induced transcription stress triggers the genome-wide degradation of promoter-bound Pol II
Source: Nat Commun. 2022 Jun 24;13:3624. doi: 10.1038/s41467-022-31329-w (PMC9232492; doi:10.1038/s41467-022-31329-w)
Supplement: Supplementary file 1 — Supplementary Information [file 41467_2022_31329_MOESM1_ESM.pdf]

Supplementary Information

# DNA damage-induced transcription stress triggers the genome-wide degradation of promoter-bound Pol II

Barbara Steurer[1,#], Roel C. Janssens[1,#], Marit E. Geijer[1], Fernando Aprile-Garcia[2], Bart Geverts[3], Arjan F. Theil[1], Barbara Hummel[2], Martin E. van Royen[3], Bastiaan Evers[4], René Bernards[4], Adriaan B. Houtsmuller[3], Ritwick Sawarkar[2, 5], Jurgen Marteijn[1,*]

# Contributed equally
* Correspondence: j.marteijn@erasmusmc.nl

[1] Department of Molecular Genetics, Oncode Institute, Erasmus MC Cancer Institute, Erasmus University Medical Center, Rotterdam, The Netherlands
[2] Max Planck Institute of Immunobiology and Epigenetics, Freiburg, Germany
[3] Department of Pathology, Optical Imaging Centre, Erasmus MC, Rotterdam, The Netherlands
[4] Oncode Institute, Division of Molecular Carcinogenesis, The Netherlands Cancer Institute, Amsterdam, The Netherlands.
[5] MRC, University of Cambridge, Cambridge, UK

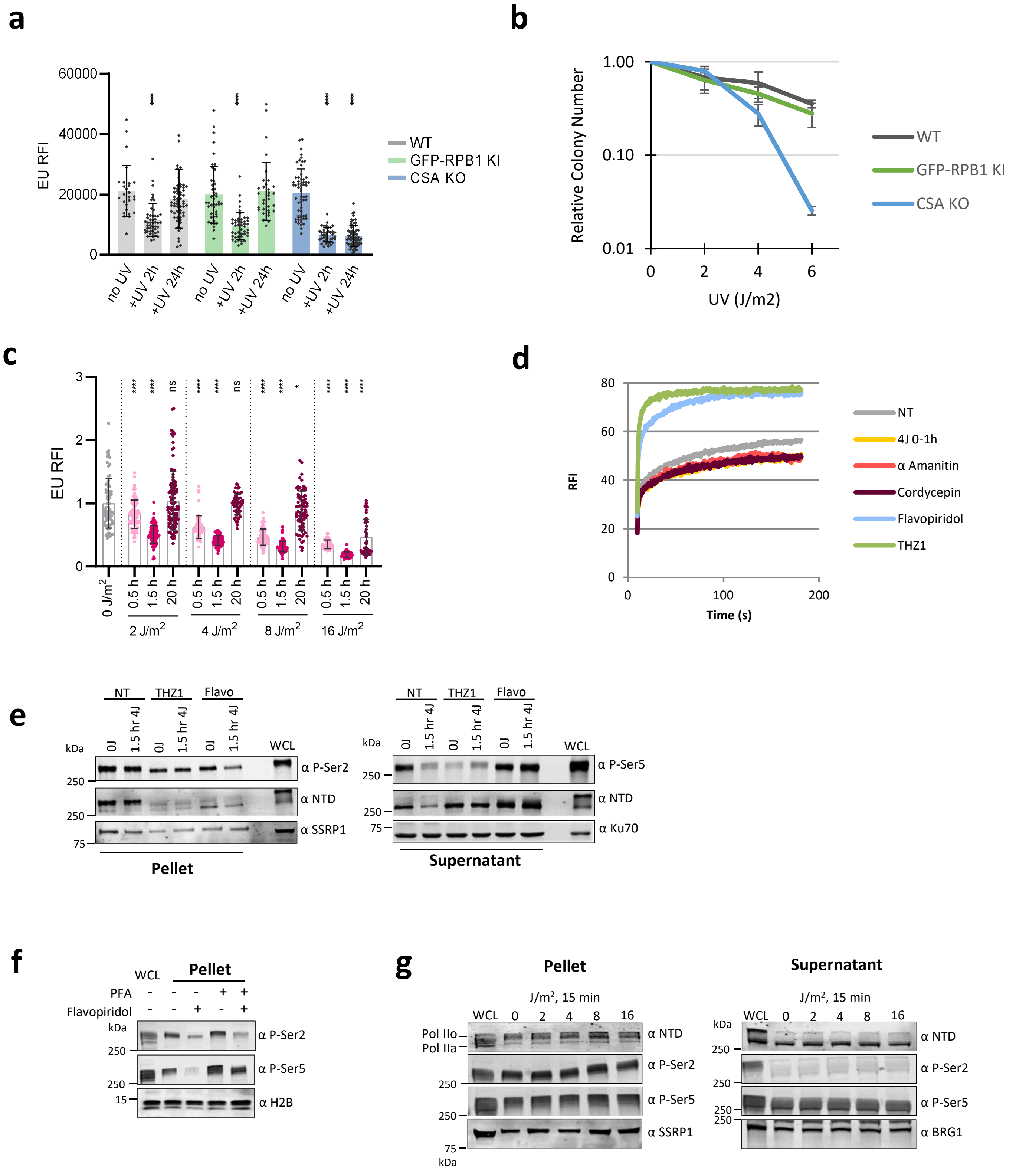


Steurer *et al*, SI

**Supplementary Figure 1**

**a** Recovery of RNA synthesis in MRC5 wild type (WT), MRC5 GFP-RPB1 knock in (KI), and MRC5 GFP-RPB1 KI CSA knock out (KO) cells measured by 1h EU pulse labelling before and 2h and 23h after irradiation with 12 J/m$^2$ of UV. EU incorporation levels were visualized by click-chemistry-based coupling to a fluorophore-labelled azide. The average nuclear fluorescence intensities (FI) of n=27,48,59,45,45,32,59,40,69 cells (left to right) were analyzed by ordinary one-way Anova using multiple comparison against the 0J condition, **** = $P < 0.0001$, non-significant differences are not indicated. Mean ± SD is plotted. **b** Right: Clonogenic survival of a single cell GFP-RPB1 knock in (KI) clone compared to parental MRC5 WT cells and a TC-NER-deficient single cell CSA KO clone. Equal numbers of cells were seeded and colony forming ability was determined in triplicate 7 days after irradiation with the indicated UV doses. Relative average colony number ± SD of 2 independent experiments is shown. **c** Recovery of RNA synthesis as described in A. of MRC5 GFP-RPB1 KI cells after irradiation with the indicated UV dose at the indicated time-points. The average nuclear fluorescence intensities (FI) of n= 78, 88, 117, 103, 68, 97, 57, 98, 92, 74, 70, 103, 51 cells (left to right) were analyzed by ordinary one-way Anova using multiple comparison against the 0J condition, **** = $P < 0.0001$, *$P < 0.05$, ns= non-significant. Mean ± SD is plotted. **d** Fluorescence Recovery after photo-bleaching (FRAP) analysis of GFP-RPB1 in non-perturbed conditions (NT) or after irradiation with 4J/m$^2$ of UV or after treatment with the elongation inhibitors α-Amanitin (100 µg/ml, 1 h) or Cordycepin (100 µM, 1h), or after incubation with THZ1 or Flavopiridol (both 2µM, 1.5h). GFP-RPB1 was bleached and fluorescence intensity was measured every 0.4 sec for 4 min, background-corrected and normalized to pre-bleach fluorescence intensity set at 100. Mean of 2 independent experiments is plotted of n=8, 16, 8, 16, 10 and 10 cells for NT, 4J, α-Amanitin, Cordycepin, Flavopiridol and THZ respectively. **e** Western blots after cellular fractionation of GFP-RPB1 knock in (KI) cells after treatment with the CDK7 inhibitor THZ1 or the CDK9 inhibitor Flavopiridol (both 1µM added 1h before UV). Ku70 was used as loading control. WCL= whole cell lysate, NTD=RPB1 N-terminal domain, P-Ser= phospho-serine. **f** Western blots after cellular fractionation of GFP-RPB1 knock in (KI) cells after treatment with the CDK9 inhibitor Flavopiridol (1µM added 90min before fractionation) with or without PFA-mediated crosslinking (1% PFA, 1 min) before fractionation. H2B was used as loading control. WCL= whole cell lysate, P-Ser= phospho-serine. **g** Representative western blots after cellular fractionation of GFP-RPB1 KI cells 15 min after irradiation with the indicated UV dosesSSRP1 and BRG1 were used as loading controls. WCL= whole cell lysate, NTD=RPB1 N-terminal domain, P-Ser=phospho-serine. Experiments plotted in (E-G) have been performed two times with similar results.

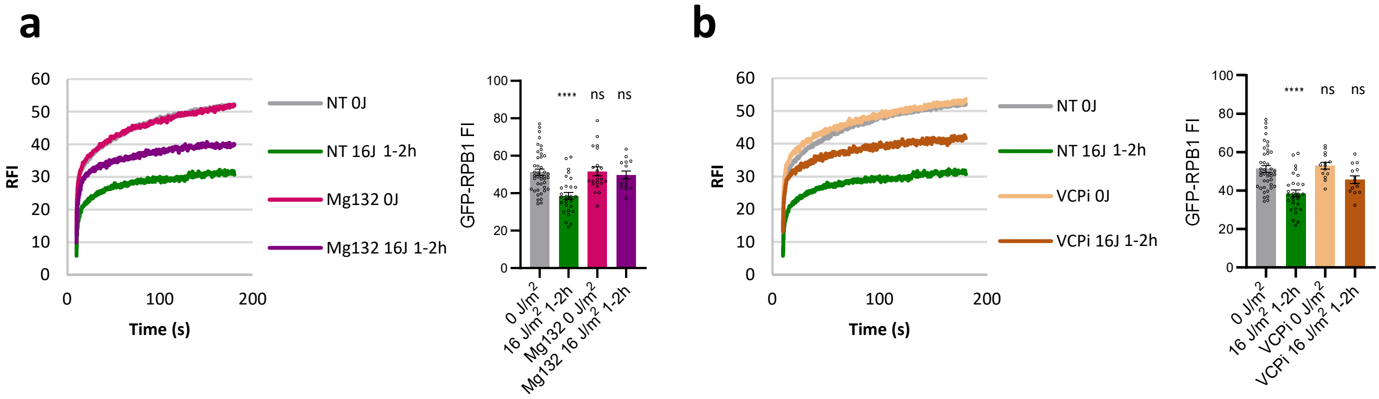


**Supplementary Figure 2**

**a,b** Left panels: Fluorescence Recovery after photo-bleaching (FRAP) analysis of GFP-RPB1 after UV irradiation with 16J/m$^2$ without and with pre-treatment for 30 minutes with (**a**) proteasome inhibitor (Mg132, 10 µM) or (**b**) VCP inhibitor (VCPi) (NMS-873, 5µM). GFP-RPB1 was bleached and fluorescence intensity was measured every 0.4 sec for 3 min, background-corrected and normalized to pre-bleach fluorescence intensity (FI), which was set to 100. RFI= relative FI. Right panel: Mean ±SD of pre-bleach GFP-RPB1 FI as a measure for Pol II protein levels in nuclei analyzed by FRAP. N=42 for NT 0J, n=29 for NT 16J 1-2h, n=22 for Mg132 0J, n=14 for Mg132 16J 1-2h, n=14 for VCPi 0J, n=14 for VCPi 16J 1-2h measured over at least 2 independent experiments and analyzed by ordinary one-way Anova using multiple comparison against the 0J condition, **** = $P < 0.0001$, ns= non-significant.

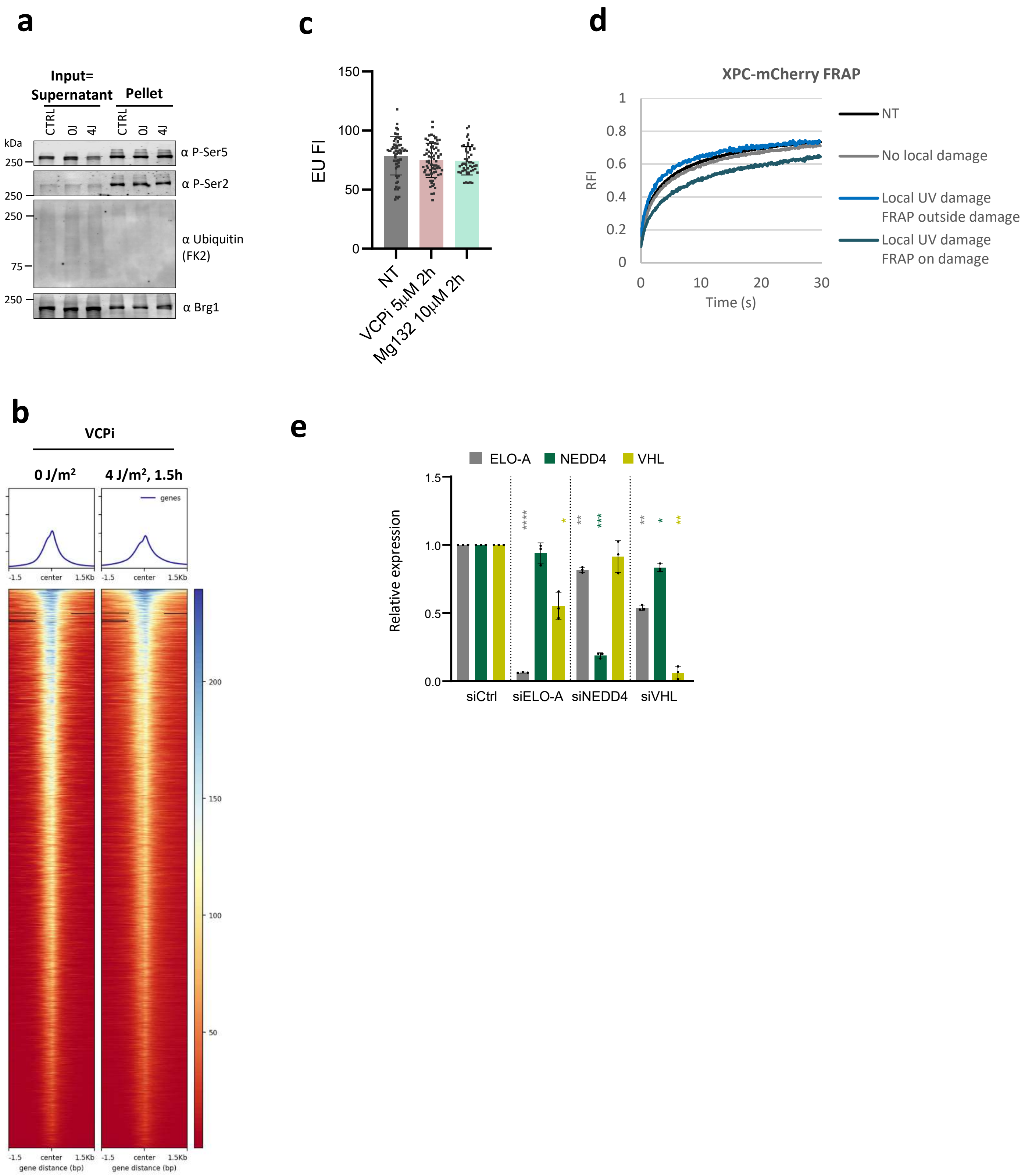

a
Input= Supernatant
Pellet
CTRL
0J
4J
kDa
250
α P-Ser5
α P-Ser2
α Ubiquitin (FK2)
75
α Brg1
c
150
100
50
0
EU FI
NT
VCPi 5μM 2h
Mg132 10μM 2h
d
XPC-mCherry FRAP
RFI
Time (s)
NT
No local damage
Local UV damage FRAP outside damage
Local UV damage FRAP on damage
b
VCPi
0 J/m²
4 J/m², 1.5h
genes
-1.5
center
1.5Kb
gene distance (bp)
200
150
100
50
e
ELO-A
NEDD4
VHL
Relative expression
siCtrl
siELO-A
siNEDD4
siVHL

**Supplementary Figure 3**

**a** Western blots stained with the indicated antibodies, after cellular fractionation of GFP-RPB1 knock in (KI) cells after the indicated treatments. Supernatant fraction was used as input for isolation of ubiquitylated proteins using tandem ubiquitin binding entities (TUBEs). BRG1 was used as loading control. P-Ser= phospho-serine. Experiment has been performed two times with similar results. **b** Pol II occupancy around the transcription start site (TSS) was analyzed by Pol II ChIP-seq on all refseq genes upon inhibition of VCP, before and 1h after 4 J/$m^2$ UV. Values were normalized to spike in controls. Heat maps are colour-scaled according to read densities form low (red) to high (blue) in units of RPM. **c** RNA synthesis levels determined by EU pulse labelling for 30 min followed by click-chemistry-based coupling to a fluorophore-labelled azide in GFP-RPB1 KI cells after the indicated treatments. The average nuclear fluorescence intensities (FI) of n= 66, 72, 52 cells (left to right) were measured in 2 independent experiments and analyzed by ordinary one-way Anova using multiple comparison against the 0J condition, non-significant differences are not indicated. Mean ± SD is plotted. **d** XPC-mCherry FRAP in untreated (NT), or after local UV irradiation through a porous filter to induce local DNA damage in cells containing local UV-damage and FRAP analysis outside the local damage (local UV damage FRAP outside damage) or FRAP of the local damage (local UV damage FRAP on damage), or in cells without local damage (no local damage). N=25 cells for NT, n= 6 for all other conditions, measured over 2 independent experiments. **e** Relative RNA expression levels measured by RT-qPCR after si-RNA-mediated knockdown of indicated genes. Mean ± SD of 3 independent replicates is shown and was analyzed by ordinary one-way Anova using multiple comparison against the siCTRL condition *$P < 0.05$; **$P < 0.01$, ***$P < 0.001$, **** $P < 0.0001$, non-significant differences are not indicated.

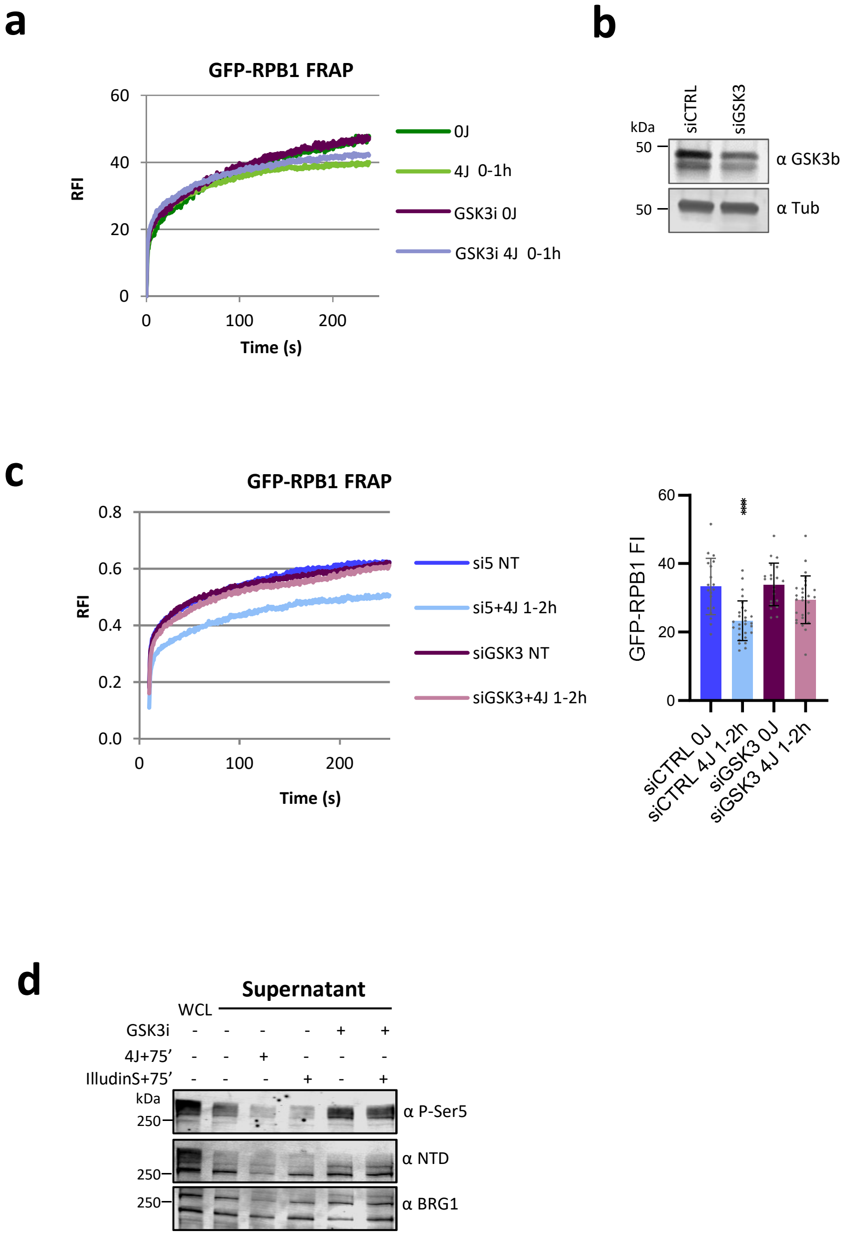
a
GFP-RPB1 FRAP
RFI
Time (s)
0J
4J 0-1h
GSK3i 0J
GSK3i 4J 0-1h
b
kDa
siCTRL
siGSK3
α GSK3b
α Tub
c
GFP-RPB1 FRAP
RFI
Time (s)
si5 NT
si5+4J 1-2h
siGSK3 NT
siGSK3+4J 1-2h
GFP-RPB1 FI
siCTRL 0J
siCTRL 4J 1-2h
siGSK3 0J
siGSK3 4J 1-2h
d
Supernatant
WCL
GSK3i
4J+75'
IlludinS+75'
kDa
α P-Ser5
α NTD
α BRG1

**Supplementary Figure 4**

**a** Fluorescence Recovery after photo-bleaching (FRAP) of GFP-RPB1 cells 0-1hr after UV irradiation with or without pre-treatment for 30 minutes with GSK3 inhibitor (CHIR-99021, 10µM). GFP-RPB1 was bleached and fluorescence intensity was measured every 0.4 sec for 4 min, background-corrected and normalized to pre-bleach fluorescence intensity (FI), which was set to 100. RFI= relative FI. N=27 cells for 0J and 4J 0-1h, n=29 cells for GSK3i 0J and GSK3i 4J 0-1h cells measured in 3 independent experiments. **b** Western Blot analysis of whole cell extracts of MRC5 GFP-RPB1 cells after siRNA transfection with either non-targeting siRNA (siCTRL) of siRNA targeting both the GSK3α and β isoforms. Tubulin is used as loading control. **c** Fluorescence Recovery after photo-bleaching (FRAP, left panel) and pre-bleach fluorescence levels (right panel) of GFP-RPB1 1-2hr after UV irradiation in cells transfected with the indicated siRNA. GFP-RPB1 was bleached and fluorescence intensity was measured every 0.4 sec for 4 min, background-corrected and normalized to pre-bleach fluorescence intensity (FI), which was set to 100. RFI= relative FI. N=19, 28, 21, 27 cells for siCtrl 0J, siCtrl 4J 1-2h, siGSK3 0J, siGSK3 4J 1-2h, respectively, measured in 3 independent experiments and analyzed by ordinary one-way Anova using multiple comparison against the 0J condition, **** = $P < 0.0001$, non-significant differences are not indicated. **d** Representative western blots after cellular fractionation of GFP-RPB1 knock in (KI) 75minutes after exposure to 100ng/ml Illudin S or without (NT), pretreated with GSK3 inhibitor treatment as indicated (GSK3i, CHIR-99021, 10 µM 30 minutes before UV). BRG1 were used as loading controls. NTD=RPB1 N-terminal domain, P-Ser= phospho-serine. Experiment has been performed two times with similar results.

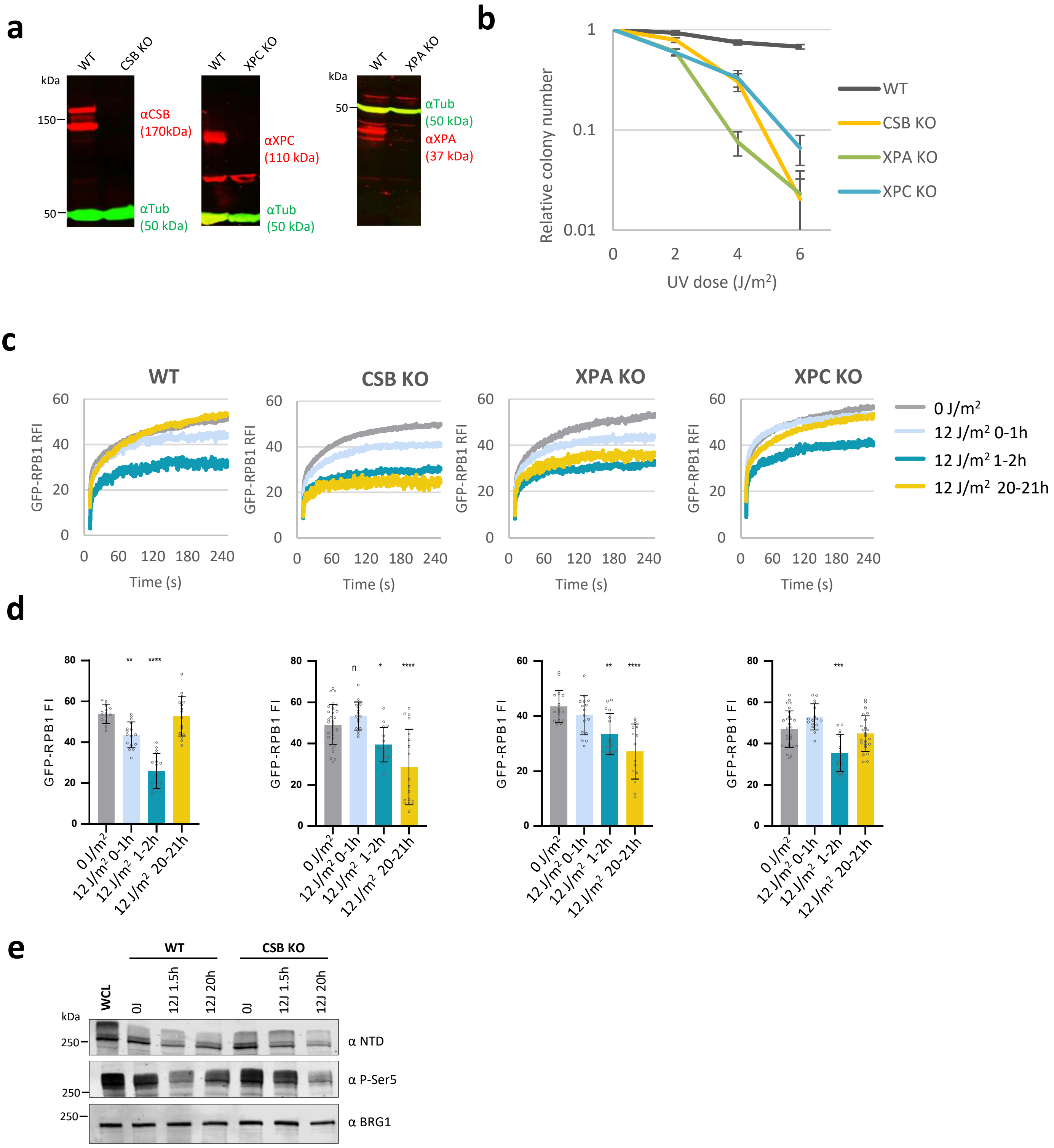


Steurer *et al*, SI

**Supplementary Figure 5**

**a** Western Blot analysis of MRC5 WT cells and MRC5 CRISPR/Cas9-mediated knock out (KO) cells of the repair proteins CSB, XPC and XPA. Respective KO cells show absence of expected bands at the indicated molecular weights confirming successfully abolished protein expression. **b** Clonogenic survival of MRC5 wild type GFP-RPB1 knock in (WT) cells compared to GFP-RPB1 KI cells with CRISPR/Cas9-mediated gene knock out of indicated DNA repair proteins. Equal numbers of cells were seeded and colony forming ability was determined in triplicate 7 days after irradiation with the indicated UV doses. Relative colony number ± SD of 2 independent experiments is shown. **c,d** As in Fig 7A and B, respectively, but after irradiation with 12 J/m$^2$. N= 15 cells for XPA KO 12J 20-21h and XPC KO 12J 1-2h, n=18 cells for CSB KO 12J 20-21h, n=23 for XPC KO 12J 20-21h, n=31 cells for XPC KO 0J, n=32 cells for CSB KO 0J, n=16 cells for all other conditions. Cells were measured over at least 2 independent experiments and pre-bleach FI levels were analyzed by ordinary one-way Anova using multiple comparison against the respective 0J condition. *$P < 0.05$; **$P < 0.01$, ***$P < 0.001$, **** $P < 0.0001$, ns= non-significant. **e** Western blots after cellular fractionation of GFP-RPB1 in wilt type cells (WT) and cells with CRISPR/Cas9-mediated CSB knockouts (KO). Fractionation was performed in non-perturbed conditions (0J) or 1.5h and 20h after irradiation with 12 J/m$^2$ (12J), only the supernatant fraction containing cytoplasmic and nucleoplasmic proteins is shown. BRG1 was used as loading control. NTD=RPB1 N-terminal domain, P-Ser= phospho-serine. Experiment has been performed two times with similar results.

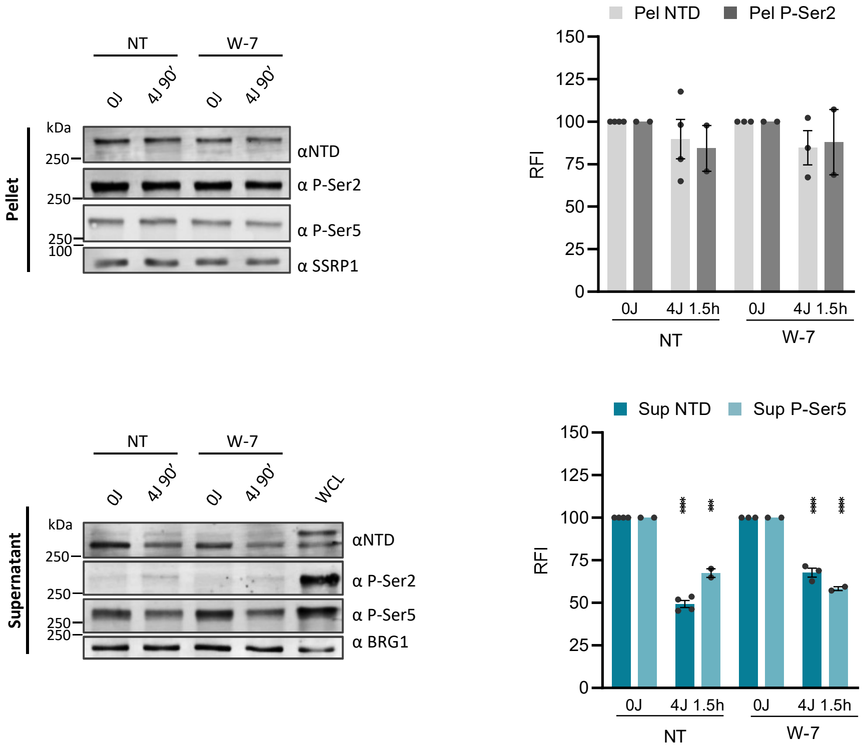


**Supplementary Figure 6**

Left: Representative western blots after cellular fractionation of non-irradiated (0J) GFP-RPB1 knock in (KI) cells or 1.5h after irradiation with 4J/m$^2$. Cells were either non-treated (NT) or pre-treated with the calmodulin antagonist W-7 for 30min before UV irradiation. Cellular fractionation was optimized to separate hyper-phosphorylated, elongating Pol IIo in the pellet from hypo-phosphorylated Pol IIa in the supernatant. SSRP1 and BRG1 were used as loading controls. WCL= whole cell lysate, NTD=RPB1 N-terminal domain, P-Ser= phospho-serine. Right: Quantification of indicated band intensities ± SD of n=2 independent western blot experiments analyzed by ordinary one-way Anova using multiple comparison against the respective 0J condition. ***$P < 0.001$, **** $P < 0.0001$, non-significant differences are not indicated.

**Supplementary Table 1**

List of antibodies, primers, siRNA and sgRNA used in this study.

| Inhibitors | Concentration | Supplier |
|---|---|---|
| THZ1 (CDK7) | 1 µM | Bioconnect |
| Cordycepin | 100µM | Sigma Aldrich |
| Flavopiridol (CDK9) | 1 µM | Sigma Aldrich |
| α-Amanitin | 100 µg/ml | Sigma Aldrich |
| Mg132 (proteasome) | 5 µM | Enzo Life Siences |
| VCP, NMS-873 | 5 µM | Selleck Chemicals |
| ATM, Ku55933 | 10 µM | Selleck Chemicals |
| ATR, VE-821 | 10 µM | Selleck Chemicals |
| p38, SB203580 | 20 µM | Cell Signaling |
| GSK3, CHIR-99021 | 10 µM | Selleck Chemicals |
| JNK, SP600125 | 20 µM | Selleck Chemicals |
| W-7 Calmodulin) | 100 µM | Merck chemicals |

| Antibodies | Dilution | clone/ ordernumber | Supplier |
|---|---|---|---|
| GFP | 1:2000 | ab290 | Abcam |
| RPB1-NTD | 1:2000 | D8L4Y | Cell Signaling |
| RPB1-P-Ser2 | 1:500 | 3E10 | Chromotek |
| RPB1-P-Ser5 | 1:500 | 3E8 | Chromotek |
| SSRP1 | 1:10000 | 10D7 | Biolegend |
| BRG1 | 1:10000 | ab110641 | Abcam |
| CSB | 1:250 | E18, sc-10459 | Santa Cruz |
| XPA | 1:1000 | GTX103168 | Genetex |
| XPC | 1:1000 | A301-121A | Bethyl |
| GSK3b Y216 | 1:1000 | 13A, 612312 | BD Bioseceinces |
| FK2 | 1:2000 | BML-PW8810 | Enzo |
| Ku70 | 1:1000 | sc-17789 | Santa Cruz |
| Rpb1 NTD | | (D8L4Y, #14958) | Cell Signaling |
| H2B | 1:1000 | 07-371 | Millipore |

**siRNAs**

| | |
|---|---|
| NEDD4 | AUGGAGUUGAUUAGAUUACAAUU |
| Elongin A | SMARTpool Dharmacon L-005143-00-0005 |
| pVHL | SMARTpool Dharmacon L-003936-00-0005 |
| Control | UGGUUUACAUGUCGACUAA |
| siGSK3 | catalog no. 6301; Cell Signaling |

**primers**

| | |
|---|---|
| RPLP1 fw | AGTAAGAACCAATCAGGCGCAGT |
| RPLP1 rev | CGCCTATACGCAGCCTCTTATGT |
| RPL3 fw | GAAGAGCGTGCGTGGAAATG |
| RPL3 rev | CGGGTCCGCTATATAAAGCCA |

**sgRNA**

| | | |
|---|---|---|
| CSB.1 | GCGAGGGCTGAACGGGATGG | *these 2 sgRNA's need to be combined to delete exon3 |
| CSB.2 | GCTTTGGAAAACTTAAGGGT | |
| XPA | GTATCGAGCGGAAGCGGCAG | |
| XPC | GCTCGGAAACGCGCGGCCGG | |
