# Supplementary material for: DNA damage-induced transcription stress triggers the genome-wide degradation of promoter-bound Pol II
Source: Nat Commun. 2022 Jun 24;13:3624. doi: 10.1038/s41467-022-31329-w (PMC9232492; doi:10.1038/s41467-022-31329-w)
Supplement: Supplementary file 2 — Reporting Summary [file 41467_2022_31329_MOESM2_ESM.pdf]

nature portfolio

Corresponding author(s): J.A. Marteijn

Last updated by author(s): May 25, 2022

Reporting Summary

Nature Portfolio wishes to improve the reproducibility of the work that we publish. This form provides structure for consistency and transparency in reporting. For further information on Nature Portfolio policies, see our Editorial Policies and the Editorial Policy Checklist.

Statistics

For all statistical analyses, confirm that the following items are present in the figure legend, table legend, main text, or Methods section.

n/a	Confirmed	
☐	☒	The exact sample size (*n*) for each experimental group/condition, given as a discrete number and unit of measurement
☐	☒	A statement on whether measurements were taken from distinct samples or whether the same sample was measured repeatedly
☐	☒	The statistical test(s) used AND whether they are one- or two-sided<br>*Only common tests should be described solely by name; describe more complex techniques in the Methods section.*
☒	☐	A description of all covariates tested
☒	☐	A description of any assumptions or corrections, such as tests of normality and adjustment for multiple comparisons
☐	☒	A full description of the statistical parameters including central tendency (e.g. means) or other basic estimates (e.g. regression coefficient) AND variation (e.g. standard deviation) or associated estimates of uncertainty (e.g. confidence intervals)
☒	☐	For null hypothesis testing, the test statistic (e.g. *F*, *t*, *r*) with confidence intervals, effect sizes, degrees of freedom and *P* value noted<br>*Give P values as exact values whenever suitable.*
☒	☐	For Bayesian analysis, information on the choice of priors and Markov chain Monte Carlo settings
☒	☐	For hierarchical and complex designs, identification of the appropriate level for tests and full reporting of outcomes
☐	☒	Estimates of effect sizes (e.g. Cohen's *d*, Pearson's *r*), indicating how they were calculated

Our web collection on statistics for biologists contains articles on many of the points above.

Software and code

Policy information about availability of computer code

Data collection	Microscopy data was obtained using commercially available Leica LAS AF software or Carl Zeiss LSM software, as indicated.
Data analysis	Data was analyzed by Leica LAS AF (version 2.7.4.10100) and LAS X (version 3.5.6.21594) software, Harmony® High Content Imaging and Analysis Software (Perkin Elmer), Carl Zeiss LSM (version 14.0.0.0), ImageJ/Fiji software (version 1.52p) and further processed in Excel (2016) and Prism (version 9).

For manuscripts utilizing custom algorithms or software that are central to the research but not yet described in published literature, software must be made available to editors and reviewers. We strongly encourage code deposition in a community repository (e.g. GitHub). See the Nature Portfolio guidelines for submitting code & software for further information.

Data

Policy information about availability of data

All manuscripts must include a data availability statement. This statement should provide the following information, where applicable:

- Accession codes, unique identifiers, or web links for publicly available datasets
- A description of any restrictions on data availability
- For clinical datasets or third party data, please ensure that the statement adheres to our policy

All the Pol II ChIP-seq data is available under GEO accession number GSE169480. Any other data are available from the corresponding author upon reasonable request.

Field-specific reporting

Please select the one below that is the best fit for your research. If you are not sure, read the appropriate sections before making your selection.

☒ Life sciences ☐ Behavioural & social sciences ☐ Ecological, evolutionary & environmental sciences

For a reference copy of the document with all sections, see nature.com/documents/nr-reporting-summary-flat.pdf

Life sciences study design

All studies must disclose on these points even when the disclosure is negative.

Sample size	No sample size calculation was performed, sample sizes are similar as to what is common in the field.
Data exclusions	No samples were excluded
Replication	xperiments were replicated two to five times, as indicated per experiment in the legends. all replication attempts were successful
Randomization	this is not applicable as our sample groups (cells growing in a dish) are not individually labeled and therefore automatically randomized when given treatment or not
Blinding	blinding was not needed as data is collected by imaging software which yield unbiased, objective measurements

Reporting for specific materials, systems and methods

We require information from authors about some types of materials, experimental systems and methods used in many studies. Here, indicate whether each material, system or method listed is relevant to your study. If you are not sure if a list item applies to your research, read the appropriate section before selecting a response.

Materials & experimental systems

n/a	Involved in the study	
☐	☒	Antibodies
☐	☒	Eukaryotic cell lines
☒	☐	Palaeontology and archaeology
☒	☐	Animals and other organisms
☒	☐	Human research participants
☒	☐	Clinical data
☒	☐	Dual use research of concern

Methods

n/a	Involved in the study	
☐	☒	ChIP-seq
☒	☐	Flow cytometry
☒	☐	MRI-based neuroimaging

Antibodies

Antibodies used

GFP 1:2000 ab290 Abcam
RPB1-NTD 1:2000 D8L4Y Cell Signaling
RPB1-P-Ser2 1:500 3E10 Chromotek
RPB1-P-Ser5 1:500 3E8 Chromotek
SSRP1 1:10000 10D7 Biolegend
BRG1 1:10000 ab110641 Abcam
CSB 1:250 E18, sc-10459 Santa Cruz
XPA 1:1000 GTX103168 Genetex
XPC 1:1000 A301-121A Bethyl
GSK3b Y216 1:1000 13A, 612312 BD Bioseceinces
FK2 1:2000 BML-PW8810 Enzo
Ku70 1:1000 sc-17789 Santa Cruz
H2B 1:1000 07-371 Millipore
Rpb1 NTD (D8L4Y) Rabbit mAb (Cell Signaling #14958) use for Pol II ChIP-seq.

Validation

Antibodies were validated as indicated on their manufacturer's website, or where validated in previous publications of our lab by siRNA/KO experiments or where checked by western blot or immunofluorescence in this manuscript, mostly with a siRNA/KO as control for specificity.
SSRP1 was validated in Dinant, Mol. Cell 2013
GFP, RPB1-NTD, RPB1-P-Ser2 and RPB1-P-Ser5 was validated in Steurer et al. 2018 PNAS
CSAB, XPA, XPC are for example validated in Suppl. Fig 5A
GSK3 is validated in 5B and S4B
H2B is validated in Mandemaker et al EMBO rep. 2018
BRG1 is validated in Ribeiro-Silva et al. Nature Comm. 2018

Eukaryotic cell lines

Policy information about cell lines

Cell line source(s)	MRC-5 (sv-40) GFP-RPB1 knock-in cells as described in Steurer, B. et al. Live-cell analysis of endogenous GFP-RPB1 uncovers rapid turnover of initiating and promoter-paused RNA Polymerase II. Proc Natl Acad Sci U S A 115, E4368-E4376 (2018). XPA KO, CSB KO and XPC KO cells in the MRC-5 (sv40) cells were generated in this study as described in the methods.
Authentication	Cells were authenticated based on morphology and GFP-RPB1 expression. XPA, CSB, XPC KO cells were authenticated on a regular basis by western blotting.
Mycoplasma contamination	All cell lines were routinely tested for mycoplasma and were all negative.
Commonly misidentified lines (See ICLAC register)	No commonly misidentified cell lines were used in this study.

ChIP-seq

Data deposition

☒ Confirm that both raw and final processed data have been deposited in a public database such as GEO.

☒ Confirm that you have deposited or provided access to graph files (e.g. BED files) for the called peaks.

Data access links *May remain private before publication.*	The following statement is included in the Data Availability section in the Main text of the manuscript: All Pol II ChIP-seq data is available under GEO accession number GSE169480.
Files in database submission	The following statement is included in the Data Availability section in the Main text of the manuscript: All Pol II ChIP-seq data is available under GEO accession number GSE169480.
Genome browser session (e.g. UCSC)	No longer applicable

Methodology

Replicates	See for details the Methods section, for each experiment two biological replicates were generated and sequenced.
Sequencing depth	See for details the Methods section, libraries were sequenced paired-end with a minimum depth of 40million.
Antibodies	See for details supplementary table 1, Protein A magnetic beads (Life Technologies, 10002D) were incubated (rotated) with 10μg of f Rpb1 NTD (D8L4Y) Rabbit mAb (Cell Signaling #14958).
Peak calling parameters	See for details the Methods section, Peak calling was done with MACS261 (model-based analysis of ChIP-seq) using “–keep-dup all,” “–nomodel,” “–extsize” and “–broad.”
Data quality	See for details the Methods section, RNA Pol II peaks ($P<1\times10{-}5$) had to overlap the TSS (from the TSS to 500bp downstream of the TSS), the reads per million (RPM) of the same region had to be at least 1, and genes had to be >2kb long and >1kb distant to any neighboring gene. If a gene had several TSSs, the highest occupied (based on RPM) was taken.
Software	See for details the Methods section, for visualization, the paired-end reads were extended to fragment size and normalized to total reads aligned (RPM) using deeptools2. Heatmaps were generated with gplots package. All browser tracks were visualized using Integrative Genome Viewer (http://software.broadinstitute.org/software/igv/).
